# Mouse strain-specific polymorphic provirus functions as *cis*-regulatory element leading to epigenomic and transcriptomic variations

**DOI:** 10.1038/s41467-021-26630-z

**Published:** 2021-11-09

**Authors:** Xuemeng Zhou, Tsz Wing Sam, Ah Young Lee, Danny Leung

**Affiliations:** 1 Division of Life Science, The Hong Kong University of Science and Technology, Clear Water Bay, Hong Kong, SAR, China; 2 Center for Epigenomics Research, The Hong Kong University of Science and Technology, Clear Water Bay, Hong Kong, SAR, China

**Keywords:** Epigenomics, Gene regulation, Mobile elements

## Abstract

Polymorphic integrations of endogenous retroviruses (ERVs) have been previously detected in mouse and human genomes. While most are inert, a subset can influence the activity of the host genes. However, the molecular mechanism underlying how such elements affect the epigenome and transcriptome and their roles in driving intra-specific variation remain unclear. Here, by utilizing wildtype murine embryonic stem cells (mESCs) derived from distinct genetic backgrounds, we discover a polymorphic MMERGLN (GLN) element capable of regulating H3K27ac enrichment and transcription of neighboring loci. We demonstrate that this polymorphic element can enhance the neighboring *Klhdc4* gene expression in *cis*, which alters the activity of downstream stress response genes. These results suggest that the polymorphic ERV-derived *cis*-regulatory element contributes to differential phenotypes from stimuli between mouse strains. Moreover, we identify thousands of potential polymorphic ERVs in mESCs, a subset of which show an association between proviral activity and nearby chromatin states and transcription. Overall, our findings elucidate the mechanism of how polymorphic ERVs can shape the epigenome and transcriptional networks that give rise to phenotypic divergence between individuals.

## Abstract (web summary)

How polymorphic elements can drive intra-species variation has not been clearly examined. Here the authors show that the polymorphic GLN endogenous retrovirus (ERV) functions as a *cis*-regulatory element for neighboring genes, leading to differences in how two mouse strains respond to stress.

## Introduction

Endogenous retroviruses (ERVs) are repetitive elements that constitute approximately 8 and 10% of human and mouse genomes, respectively^1,2^. They are relics of exogenous viruses that invaded the germline genomes of their hosts^3^. A full-length inserted sequence or provirus consists of two identical long terminal repeats (LTRs) flanking viral genes, essential for their replication and amplification by retrotransposition^4^. While some elements remain intact throughout evolution, the majority of ERVs in mammalian genomes have been reduced to solo LTRs or fragments of the original^5^. These elements can disrupt proper transcriptional regulation and induce genome instability in the host^6^. Therefore, defense mechanisms, including epigenetic pathways, work to repress ERVs and mitigate their deleterious potentials. In fact, proviral reactivation coupled with epigenetic dysfunction has been described in a number of human diseases^7–9^. Interestingly, a subset of ERVs can escape silencing by participating in normal molecular functions in the host. They contribute new protein coding sequences or donate *cis*-regulatory elements for regulating gene expression in distinct cell types^10–12^. For instance, mammalian Syncytin proteins are encoded by ERV *env* genes, which mediate cell fusion in the placenta^11,13^. In recent studies, ARC, which is necessary for synaptic maturation and cognition, was discovered to also originate from ERVs^14,15^. Moreover, HERV-H and MERVL elements function as long non-coding RNA (lncRNA) and *cis*-regulatory elements to maintain cell identity in human and mouse stem cells, respectively^12,16,17^.

While human ERVs (HERVs) are commonly believed to be fixed in the genome, recent studies indicated that members of the HERV-K HML-2 subfamily still possess the ability to retrotranspose^18–23^. In the mouse genome, a much larger proportion of murine ERVs retain their retrotransposition activity. The copy-and-pasting of a subset of elements is responsible for approximately 10–12% of all germline mutations^24^. IAP and ETn/MusD are two ERV-K subfamilies with many documented polymorphic integrations. A targeted genomic assessment estimated that at least 60% of IAP and 25% of ETn/MusD elements differ among four laboratory mouse strains^25^. Moreover, several studies have reported the association between polymorphic ERVs and the dysregulation of adjacent genes^26–29^. For example, an intronic IAP inserted in the C57BL/6 strain triggered the premature termination of *Slc15a2*^30^. Likewise, a pair of MusD insertions in the upstream and intronic regions of *Fbxw4* were discovered to generate loss of function alleles in a dactylaplasia mouse model^31–33^. Also, other elements, including MLV and ERVB4, can influence neighboring gene expression^34–37^. Notably, MMERGLN (abbreviated as GLN), belonging to the class I ERVs (ERV-1), is capable of generating infectious viral particles^38^. These ERV-derived virions can potentially spread to neighboring cells by binding to SLC19A1 receptors^39^. Given the possible damaging consequences, epigenetic modifiers, such as SETDB1, function to repress these proviruses^40^. Interestingly, GLN expression was induced in mouse liver while under genotoxic stress^41^, which regulated cellular proliferation through an unknown mechanism. The transcript was proposed to play a physiological function, but the precise model had not been elucidated. Taken together, new retrotransposition events in the mouse genome can affect normal gene expression and lead to aberrant phenotypes. However, the extent of polymorphic proviruses in wildtype (WT) animals and their impact on the epigenome and transcriptome have not been examined.

To delineate the effects of polymorphic ERVs, we analyze murine embryonic stem cells (mESCs) from two commonly used laboratory mouse strains. We discover a full-length GLN provirus that integrated near the *Klhdc4* gene in the C57BL/6 and CBA/J strains but is absent in some other genetic backgrounds, including the 129S strains. The insertion functions as a *cis*-regulatory element, causing upregulation of *Klhdc4* expression and enrichment of active histone modifications at nearby loci. Total RNA-seq analysis reveals that this polymorphic GLN contributes to strain-specific transcriptomic features involving the ATF4 transcription factor, associated with the differential stress responses among mouse strains. Subsequently, we define an extensive list of potential polymorphic ERVs in the genomes of both lines and uncover a subset with epigenetic signatures of *cis*-regulatory elements. Regions adjacent to these elements are associated with concordantly higher transcription and enrichment of active histone modifications. Overall, our results illustrate the mechanism by which naturally occurring polymorphic ERVs could regulate strain-specific transcriptional networks and chromatin states. In addition, this phenomenon is far more ubiquitous than previously appreciated. These findings shed light on how polymorphic transposons can introduce new *cis*-regulatory elements that give rise to inter-individual transcriptomic and phenotypic differences.

## Results

### Polymorphic GLN integration is associated with differential epigenetic states and gene expression

**Fig. 1:**
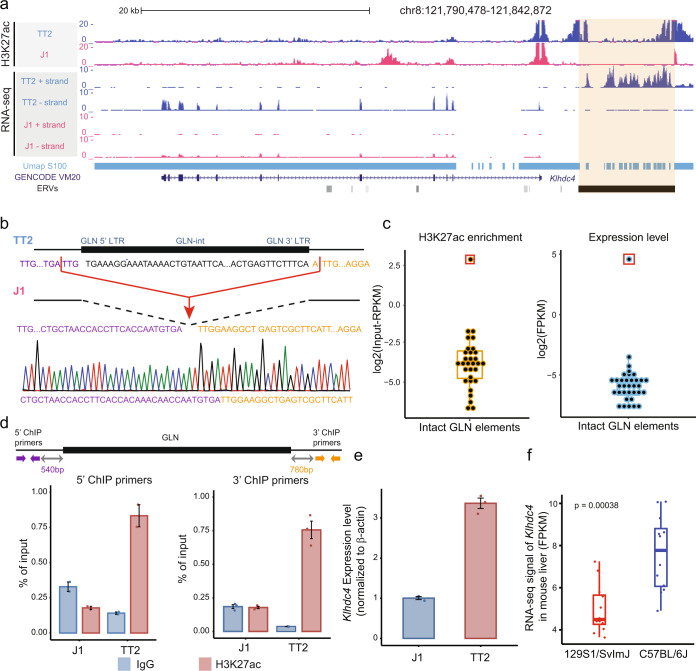
Polymorphic GLN integration is associated with the differential epigenomic states and gene expression between distinct genetic backgrounds. **a** Genome browser screenshot illustrates the presence of a polymorphic GLN element (orange shading) in TT2 and not J1 genomes. The integration is associated with higher H3K27ac enrichment and transcription of both the polymorphic GLN element (on + strand) and the *Klhdc4* gene (on − strand) in TT2 cells. ChIP-seq datasets are shown as input-subtracted Reads Per Kilobase per Million reads (Input−RPKM) values and RNA-seq datasets are displayed as Reads Per Million (RPM). The Umap track shows the mappability calculated from single end 100 bp reads. Particular regions within the GLN element have relatively poor mappability. **b** Schematic illustrates the GLN insertion in the two cell lines and the Sanger sequencing result of the locus in the J1 genomic DNA. LTR: Long Terminal Repeat. **c** Boxplots of the H3K27ac ChIP-seq (left) and RNA-seq signals (right) of all the intact GLN elements in the mm10 reference assembly (*n* = 40). The polymorphic GLN element is marked with a red box. ChIP-seq signals are shown as log2-transformed Input−RPKM values and RNA-seq signals are displayed as log2-transformed Fragments Per Kilobase per Million reads (FPKM). The center and the bounds of boxes refer to the median and quartile of all data points, respectively. The minima and maxima of boxplots indicate quartile 1 − 1.5 × Interquartile range and quartile 3 + 1.5 × interquartile range, respectively. **d** ChIP-qPCR results demonstrate the increased H3K27ac enrichment at regions 5′ (left) and 3′ (right) of the polymorphic GLN element in TT2 compared to J1 mESCs. Positions of primers are indicated in the top schematic. IgG is included as a negative control. Error bars reflect standard deviations with the centers indicating the means of three technical replicates. **e** RT-qPCR results validated increased *Klhdc4* expression in TT2 compared to J1. Error bars reflect standard deviations with the centers indicating the means of three technical replicates. **f** Boxplot shows the expression level in FPKM of *Klhdc4* in the liver of two different mouse strains^44^ (*n* = 12). The center and bounds of boxes refer to the median and quartile of all data points, respectively. The minima and maxima of boxplots indicate Quartile 1 − 1.5 × interquartile range and Quartile 3 + 1.5 × interquartile range, respectively. *P* value is calculated by two-tailed *T*-test.

Previous studies have reported the existence of polymorphic ERVs between distinct mouse strains^18,25^. However, the mechanism of how these proviruses affect gene expression and whether they contribute to strain-specific molecular networks remains unclear. To investigate this phenomenon, we utilized two mouse mESC lines from different genetic backgrounds: J1 and TT2, derived from the 129S4/SvJae strain and C57BL/6×CBA/J F1 embryos, respectively. We came across a structural variant defined by the Mouse Genomes Project^42^ (chr8: 121,832,845–121,841,264 in mm10 genome assembly), which was in fact a full-length polymorphic GLN (MMERGLN) element present in C57BL/6 and CBA/J but not in 129S genome assemblies (Fig. 1a). PCR and Sanger sequencing were performed to validate its presence in TT2 and absence in J1 cells (Fig. 1b and Fig. S1a, S1b). Notably, this element was highly expressed and enriched with histone H3 lysine 27 acetylation (H3K27ac) in TT2 (Fig. 1a). Intriguingly, the polymorphic element proved to be the exception, as it was the only intact GLN element in the entire genome that was highly transcribed and enriched with active histone marks (Fig. 1c and Fig. S1c). All other GLN elements harbored significantly higher enrichment of the repressive histone modification, H3 lysine 9 trimethylation (H3K9me3), in TT2 cells (Fig. S1d). Phylogenetic analysis of all intact GLN proviruses revealed this polymorphic element to be a relatively recent integration (Fig. S1e), consistent with the concept that younger ERVs accumulated fewer mutations and had greater chances to avoid epigenetic repression.

As H3K27ac is a mark of active *cis*-regulatory elements^43^, including promoters and enhancers, we next examined the effects of the polymorphic GLN on adjacent regions. ChIP-seq and stranded total RNA-seq analyses of J1 and TT2 cells revealed that the proviral insertion was associated with increased H3K27ac enrichment at neighboring loci and upregulated expression of a nearby gene, Kelch-domain containing 4 (*Klhdc4*) (Fig. 1a). It should be noted that due to its repetitive nature, the polymorphic GLN element had poor mappability, which impacted the unique alignment of short next-generation sequencing (NGS) reads (Fig. 1a). As such, we carried out paired-end sequencing for all NGS-based assays to improve its detection. The differential H3K27ac enrichment and transcription levels were confirmed by ChIP-quantitative PCR (qPCR) and RT-qPCR, respectively (Fig. 1d, e). Notably, *Klhdc4* showed a >3-fold increase in expression and concomitant elevated protein levels in TT2 compared to J1 (Fig. 1e and Fig. S1f). These results suggested that the insertion of the polymorphic GLN may have introduced a novel *cis*-regulatory element into the C57BL/6 genome.

To determine whether this phenomenon occurred in other cell types and in other mouse colonies, we analyzed publicly available RNA-seq datasets of tissues from C57BL/6 and 129S1/SvJ strains. To avoid potential confounding factors of cell type heterogeneity, we first selected datasets from a representative tissue type with high *Klhdc4* expression. Given GLN-derived lncRNA had been proposed to function in the liver^41^, we focused on liver transcriptomes (*n* = 12)^44^. In line with our mESC results, *Klhdc4* transcripts were significantly more abundant in C57BL/6 compared to 129S1/SvJ mice (Fig. 1f), implying that it was a strain-specific feature and not limited to mESCs. We then integrated additional datasets from different tissue types. While *Klhdc4* and the polymorphic GLN were expressed in many tissue types of C57BL/6 animals (Fig. S1g), their transcript levels varied greatly. Of note, their expression levels were significantly correlated (*R* = 0.932 (Pearson); *P* = 0.0003), further supporting the hypothesis that this polymorphic GLN served as a *cis*-regulatory element for *Klhdc4*.

### Deletion or silencing of polymorphic GLN leads to downregulation of *Klhdc4* through loss of *cis*-regulatory function

**Fig. 2:**
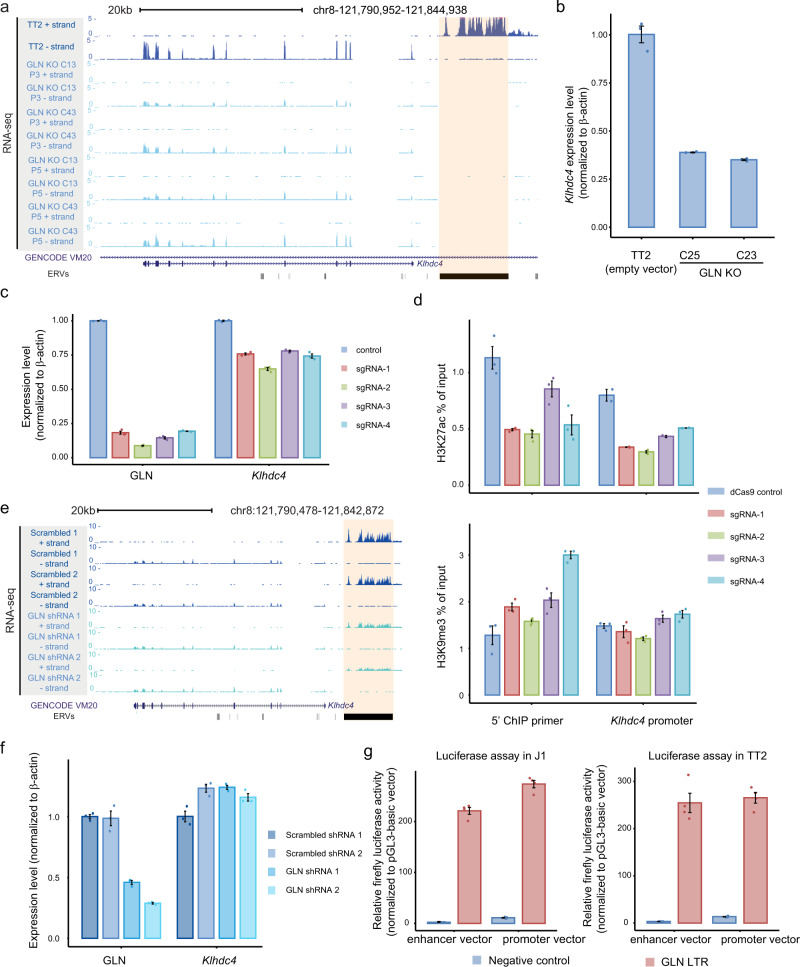
Deletion or silencing of polymorphic GLN leads to downregulation of *Klhdc4* through loss of *cis*-regulatory function. **a** A genome browser screenshot of stranded total RNA-seq illustrates the reduced expression of *Klhdc4* upon deletion of the GLN element (orange shading). Two independent GLN KO clones (C13 and C43) of different passage numbers (P3: passage 3; P5: passage 5) and two WT TT2 replicates were analyzed. RNA-seq datasets are displayed as reads per million (RPM). **b** RT-qPCR result shows downregulated expression of *Klhdc4* in both GLN KO clones at early passage compared to vector only control. Expression is normalized to the *β-actin* gene. Error bars reflect standard deviations with the centers indicating the means of three technical replicates. **c** RT-qPCR result shows concomitantly decreased expression of GLN and *Klhdc4* upon CRISPRi-mediated silencing. Normalized expression, relative to *β-actin*, for cells transduced with each sgRNA is compared to vector only control. Error bars reflect standard deviations with the centers indicating the means of three technical replicates. **d** ChIP-qPCR of sgRNA-transduced and control cells indicates a loss of H3K27ac (top) at both 5′ region of GLN and the *Klhdc4* promoter upon CRISPRi repression. However, a subtle increase of H3K9me3 (bottom) is only detected at the GLN element. Error bars reflect standard deviations with the centers indicating the means of three technical replicates. **e** A genome browser screenshot of stranded total RNA-seq datasets illustrates shRNA-mediated knockdown of GLN (orange shading) does not affect *Klhdc4* expression. RNA-seq datasets are displayed as reads per million (RPM). **f** RT-qPCR validates the decreased normalized expression, relative to *β-actin*, of GLN and unchanged expression of *Klhdc4* in GLN KD cells compared to scrambled shRNA controls. Error bars reflect standard deviations with the centers indicating the means of three technical replicates. **g** Luciferase assay results show that the LTR region of the polymorphic GLN has *cis*-regulatory function in J1 (left) and TT2 (right) cells. The *y-*axes represent relative firefly luciferase activity normalized to basic vector (with no promoter and enhancer activity). Random GFP sequence with no *cis*-regulatory activity is included as negative control. Error bars reflect standard deviations with the centers indicating the means of four technical replicates.

To functionally assess the regulatory relationship between the polymorphic GLN element and *Klhdc4* expression, we utilized CRISPR-Cas9 to knockout the provirus (GLN KO) in TT2 cells and performed RNA-seq on WT and mutant clones at different passage numbers (Fig. 2a and Fig. S2a, S2b). The homozygous deletion of the polymorphic GLN was detected in the RNA-seq datasets and validated by PCR (Fig. 2a and Fig. S2a). Coupled with the proviral deletion, *Klhdc4* transcript and protein levels were significantly reduced (Fig. 2a, b and Fig. S2c, S2d).

Having demonstrated that the loss of the polymorphic GLN affects *Klhdc4* expression, we next examined the molecular mechanism. We hypothesized that the polymorphic GLN served as an enhancer for *Klhdc4*. To test this model, we utilized CRISPR interference (CRISPRi) to repress the polymorphic GLN by altering its chromatin state. We designed a series of single guide RNAs (sgRNAs) that targeted the 5′ region of the provirus (Fig. S2e) and was able to efficiently repress its expression (>80% reduction relative to vector only control) (Fig. 2c). Given that this polymorphic GLN was the only derepressed GLN element in TT2 cells, the effects of targeting other GLN elements should not substantially alter their transcriptional and chromatin states. Consistent with our model, a moderate reduction of *Klhdc4* expression was detected in TT2 cells transduced with each of the four sgRNAs (Fig. 2c). As mammalian genes mostly possessed multiple redundant enhancers^45–48^, inhibition of a single *cis*-regulatory element was expected to only yield modest change. Moreover, heterogeneity among the transfected pool of mESCs could also contribute to the different degrees of *Klhdc4* downregulation as compared to GLN KO clones. Next, we further inspected the epigenetic states by measuring H3K27ac and H3K9me3 enrichment at the 5′ end of the polymorphic GLN and the *Klhdc4* promoter by ChIP-qPCR. As expected, decreased H3K27ac enrichment was observed at both the GLN flanking region and the *Klhdc4* promoter (Fig. 2d and Fig. S2f). Due to the repetitive nature of the GLN-LTR, we were unable to specifically target the polymorphic element by PCR. Nevertheless, we detected a subtle increase of H3K9me3 enrichment proximal to the 5′ end of the polymorphic GLN, which likely reflected the deposition of the repressive mark within the element. The varied enrichment levels among cells transduced with different sgRNAs may result from differing sgRNA expression levels or number of copies per cell. Importantly, the *Klhdc4* promoter showed no gain of H3K9me3 enrichment upon GLN silencing, confirming that the reduced expression did not result from the unintended deposition of H3K9me3 at the TSS (Fig. 2d and Fig. S2f). Thus, we concluded that epigenetic silencing of the polymorphic GLN element led to the reduction of *Klhdc4* expression and of active marks enrichment at its promoter.

As ERV-derived lncRNAs have been reported to impact transcription^12^, we asked whether the RNA transcript from the polymorphic GLN was necessary for its regulatory role. We utilized shRNAs to knockdown (KD) the polymorphic GLN in WT TT2 cells and analyzed transcriptional changes by RT-qPCR and RNA-seq. While GLN was reduced by 60–80%, *Klhdc4* expression remained unaltered (Fig. 2e, f), indicating that the GLN transcript has no role in this function. Taken together, these results further supported the putative *cis*-regulatory functions of the polymorphic GLN element. To functionally validate its capability to perform as a *cis*-regulatory element, luciferase assays were conducted. The GLN-LTR sequence or a size-matched negative control region was cloned either upstream or downstream to a luciferase transgene (Fig. S2g). Constructs were transfected into both TT2 and J1 mESCs for analysis. The GLN-LTR sequence yielded dramatically higher promoter activity compared to the negative control (Fig. 2g). This was in line with the endogenous polymorphic GLN element being highly transcribed from its LTR. Moreover, the GLN-LTR also showed significant enhancer activity (Fig. 2g). These observations were consistent between both cell lines and indicated that the GLN-LTR was sufficient to function as both promoter and enhancer. Sequence analysis also revealed that the GLN-LTR region harbored binding motifs of several transcription factors (TFs) including P53, PBX1, and TST1 (Supplementary Data 1). Taken together, these results further supported the model that the polymorphic GLN element functioned as a *cis*-regulatory element for the *Klhdc4* gene.

### GLN element can direct nearby epigenetic marks and transcription in a position-dependent manner

**Fig. 3:**
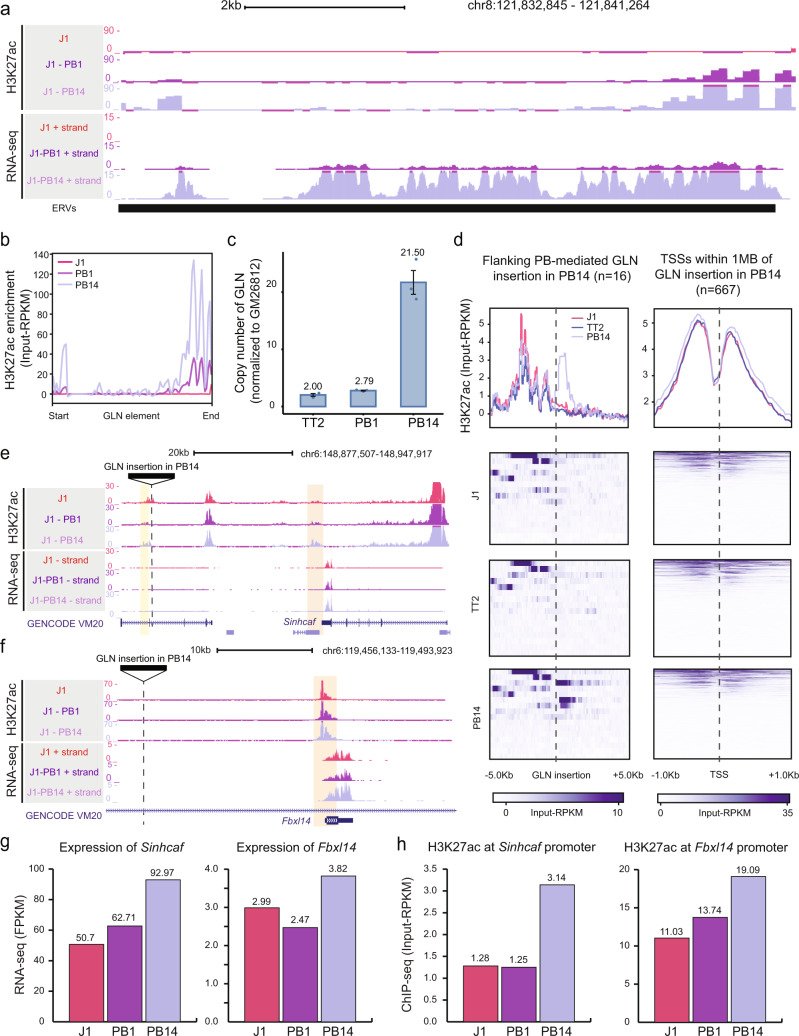
GLN integrations can shape nearby epigenetic and transcriptional states in a position-dependent manner. **a** A genome browser screenshot shows the H3K27ac (Input-RPKM) and RNA-seq (RPM) signals from WT J1 and 2 PiggyBac clones: clone 1 (PB1) and clone 14 (PB14), aligning to the polymorphic GLN locus. As new integrations are identical to the endogenous element, reads from PB-mediated insertions are attributed to the polymorphic GLN. As expected, no signal is detected in J1 cells. Aggregated increase of both H3K27ac and transcription are detected in both PB1 and PB14 mESCs. **b** Aggregation plot of H3K27ac ChIP-seq (Input-RPKM) signal at polymorphic GLN sequence demonstrates the quantitative increase in activity in PB1 and PB14 clones. **c** Copy number qPCR reveals different copy numbers, relative to single copy gene (GM26812), of GLN insertions in PB1 and PB14 genomes. TT2 genomic DNA (gDNA) is used as a positive control. Error bars reflect standard deviations with the centers indicating the means of three technical replicates. **d** Aggregation plot and heatmap show H3K27ac ChIP-seq signal (Input-RPKM) within ±5 kb of validated GLN insertions (*n* = 16) (left) and transcription start sites (TSSs) within 1 MB of GLN insertions (*n* = 667) (right) in PB14 compared to J1, and TT2. While a subset of new elements gain activity, others are not enriched with H3K27ac. **e** A genome browser screenshot of a PB-mediated insertion site in PB14 that is associated with increased H3K27ac signal at both the integration (yellow shading) and distal regions (orange shading). The nearby *Sinhcaf* gene is upregulated in PB14 cells. **f** Another example of a novel PB-mediated insertion in PB14 with no H3K27ac signal at the integration, concordant with increased transcription of *Fbxl4* and slight increase of H3K27ac signal at its promoter region (orange shading). **g** Bar charts indicate higher expression level (FPKM) of *Sinhcaf* (left) and *Fbxl14* (right) in PB14 than WT J1 and PB1. **h** Bar charts show the higher H3K27ac enrichment (Input−RPKM) on the TSS peaks of *Sinhcaf* (left) and *Fbxl14* (right) (orange shading in **e** and **f**) in PB14 than WT J1 and PB1 cells.

To examine whether the GLN element can exert similar effects at different genomic positions, we employed the PiggyBac transposon system (PB) to insert the intact GLN sequence into random positions within the J1 genome. We analyzed two clones with distinct integration patterns by H3K27ac ChIP-seq and RNA-seq. As new integrations were identical to the endogenous polymorphic GLN sequence, NGS reads alignment to mm10 genome assembly would attribute all corresponding signals to the endogenous locus. Given that the J1 genome lacked said element, all reads mapping to the polymorphic GLN must be derived from PB-mediated new insertions. Remarkably, the two PB clones (PB1 and PB14) showed different degrees of H3K27ac enrichment and RNA-seq signal on the GLN element (Fig. 3a, b), suggesting at least a proportion of new integrations were epigenetically and transcriptionally active. As expected, no signal was detected in J1 (Fig. 3a, b). The difference in signals between the PB clones was in part due to the inserted copy numbers. Indeed, copy number qPCR detected more integrations in PB14 than PB1 (Fig. 3c). To characterize the nearby changes, we first pinpointed the genomic locations of new insertions by inverse PCR and Sanger sequencing. We experimentally validated 5 loci in PB1 and 16 loci in PB14 (Fig. S3a and Table S1). Aggregated analysis showed a higher H3K27ac ChIP-seq signal at the regions adjacent to novel insertions (Fig. 3d and Fig. S3b). Furthermore, moderate increases of H3K27ac were detected at the promoters of genes within 1MB of these new GLN integrations sites (Fig. 3d and Fig. S3c). Analyzing individual integrations, we found that only a subset of loci displayed a dramatic gain of H3K27ac signal at the adjacent sequences (Fig. 3d and Fig. S3b–f), suggesting the activity of newly inserted GLN relied on neighboring sequence or chromatin states. A proportion of these elements were also associated with increased expression of nearby transcripts. For instance, we discovered a PB-mediated insertion upstream of the *Sinhcaf* gene in the PB14 genome. This element was associated with substantially higher H3K27ac signal at several nearby loci (Fig. 3e, h), as well as upregulated expression of *Sinhcaf* (Fig. 3e, g). Moreover, we noted another insertion near the *Fbxl14* gene, which was associated with the significant transcriptional upregulation but only mild increase of H3K27ac (Fig. 3f, g, h). This was perhaps because the gene promoter was already highly enriched with active marks in WT cells. Taken together, we surmised that newly inserted GLN proviruses could mediate epigenomic and transcriptomic changes of the neighboring regions in a position-dependent way. Features including epigenetic modifications, chromatin accessibility, and higher-order chromatin structures of the integration sites may all be determining factors and require further study.

### The polymorphic GLN element contributes to strain-specific transcriptomic patterns

**Fig. 4:**
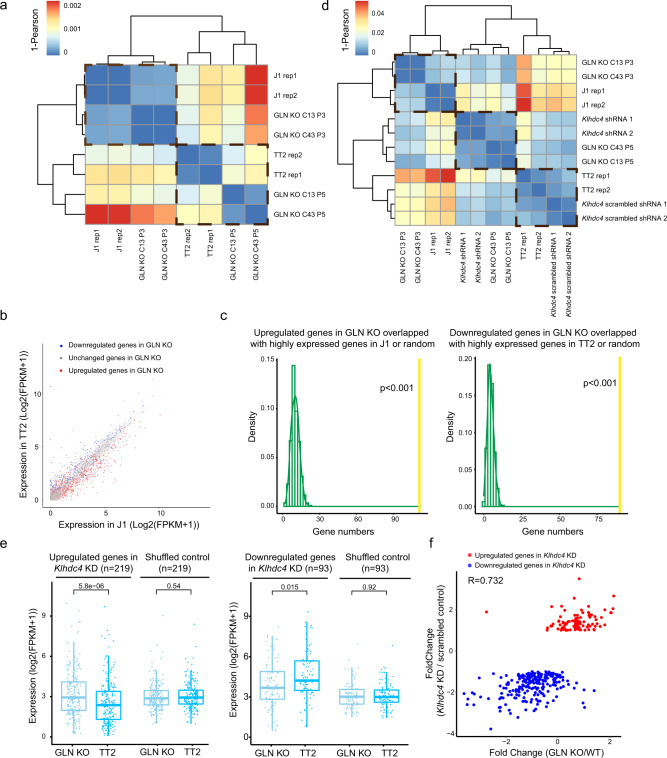
The polymorphic GLN element contributes to strain-specific transcriptomic patterns. **a** Hierarchical clustering heatmap indicates closer distance between transcriptomes of early-passage GLN KO clones (P3: passage 3) and J1 cells. Whereas later-passage clones (P5: passage 5) clustered closer to TT2. The color bar is adjusted based on correlation score (1−Pearson correlation coefficient) of the top variable genes among all samples (*n* = 1,000). **b** Scatter plot shows the gene expression levels (log2(FPKM + 1)) in WT TT2 and J1 mESCs. The downregulated and upregulated genes upon GLN KO are highlighted in blue and red, respectively. **c** Histograms/density curves indicate the distribution of overlap between randomly selected genes (selected 1,000 times) and upregulated (*n* = 647) (left) and downregulated (*n* = 310) (right) genes upon GLN KO. The yellow lines show the actual number of upregulated (*n* = 111) and downregulated (*n* = 90) genes that are commonly defined with significantly higher expression in J1 (*n* = 815) and TT2 (*n* = 843), respectively. One-tailed *P* values without multiple comparison adjustment are obtained via non-parametric bootstrapping. **d** Hierarchical clustering heatmap indicates closer distance between transcriptomes of *Klhdc4* KD and GLN KO mESCs. The color scale is adjusted based on correlation score (1−Pearson correlation coefficient) of the top variable genes among all samples (*n* = 2,000). **e** Boxplots demonstrate the expression levels of dysregulated genes upon *Klhdc4* KD and shuffled control genes in WT TT2 and GLN KO cells. The expression levels are shown as log2(FPKM + 1). The expression level of shuffled control is derived from the average of 50 times of shuffling. *P* value is calculated by Wilcoxon test. **f** Scatter plot compares the fold change of dysregulated genes upon *Klhdc4* depletion in GLN KO and *Klhdc4* KD cells, relative to their corresponding controls (WT TT2 and scrambled control, respectively). A positive correlation is observed with Pearson correlation coefficient of 0.732. Fold changes are calculated from FPKM of treated cells/control cells.

To comprehensively characterize the effects of the polymorphic GLN element on the transcriptome, we analyzed RNA-seq datasets of GLN KO cells at different culturing passage numbers and of WT mESCs. Global transcriptomic analysis by both hierarchical clustering and principal components analysis (PCA) revealed that early-passage KO clones clustered with J1 (Fig. 4a and Fig. S4a), suggesting that the polymorphic GLN element may be a substantial contributor to the strain-specific transcriptomic differences. Surprisingly, later-passage KO clones clustered more closely with WT TT2 (Fig. 4a and Fig. S4a). This reversion after several rounds of cell division was potentially due to cellular adaption and enhancer redundancy, analogous to the previously described Temp enhancers and shadow enhancers^47,49^. Consistent with global analysis, *Klhdc4* expression was mildly elevated in late passage versus early-passage GLN KO cells (Fig. S4b). Concomitantly, late-passage GLN KO cells showed a reduction of H3K27ac levels at the proximal regions and increased enrichment at distal regions compared to WT TT2 (Fig. S4c). Notably, these distal peaks with increased H3K27ac in late-passage GLN KO cells also had higher signal in J1 compared to WT TT2 mESCs (Fig. S4c), concordant with being potential redundant enhancers.

Next, we defined significantly upregulated (*n* = 647) and downregulated (*n* = 310) genes in GLN KO cells (fold change > 2, adjusted *P* value < 0.01). Consistent with clustering analyses, the majority of these upregulated genes displayed higher expression in J1 compared to TT2 cells. Inversely, most of the downregulated genes in GLN KO cells were more highly transcribed in TT2 (Fig. 4b). Gene Set Enrichment Analysis (GSEA) yielded similar findings (Fig. S4d). Furthermore, when comparing the transcriptomes of WT TT2 and J1 cells, 843 and 815 genes were defined with significantly higher expression in J1 and TT2, respectively (fold change > 2, adjusted *P* value < 0.01). Among them, 111 genes with higher expression in J1 were upregulated in GLN KO cells, while 90 genes with higher expression in TT2 were downregulated upon GLN deletion (Fig. 4c). The overlap of these definitions was significantly higher than that expected from randomly selected loci. Collectively, these findings revealed that deletion of the polymorphic GLN in the TT2 genome produced transcriptomic changes that resemble J1 cells.

To determine the contribution of *Klhdc4* downregulation to the overall transcriptome changes observed in GLN KO cells, we conducted shRNA-mediated *Klhdc4* KD in TT2 cells. The KD efficiency was approximately 70–80% (Fig. S4e), which was comparable to the reduction noted in GLN KO cells (Fig. 2b). RNA-seq analysis of the *Klhdc4* KD and scrambled control cells yielded 219 upregulated genes and 93 downregulated genes (fold change > 2, adjusted *P* value < 0.01). Hierarchical clustering revealed closer distance between *Klhdc4* KD and GLN KO cells, while the scrambled control was closer to WT TT2 (Fig. 4d). GSEA and comparison of commonly dysregulated genes both demonstrated analogous transcriptomic patterns of *Klhdc4* KD and GLN KO cells (Fig. S4f, S4g and Supplementary Data 2). Quantitative analysis of the expression levels confirmed that genes dysregulated upon *Klhdc4* KD exhibited significant concordant changes in GLN KO cells (Fig. 4e). In addition, the degrees of transcriptional change (fold change) for these genes were positively correlated in the two cell lines (Fig. 4f). For example, the *Scarb2* gene was similarly upregulated upon *Klhdc4* KD and in GLN KO cells and showed higher expression in J1 compared to TT2 mESCs (Fig. S4h, S4i). These results suggested that the reduction of *Klhdc4* expression was in part responsible for the altered transcriptomic patterns of GLN deletion.

### Polymorphic GLN regulates transcriptional network through *Atf4*

**Fig. 5:**
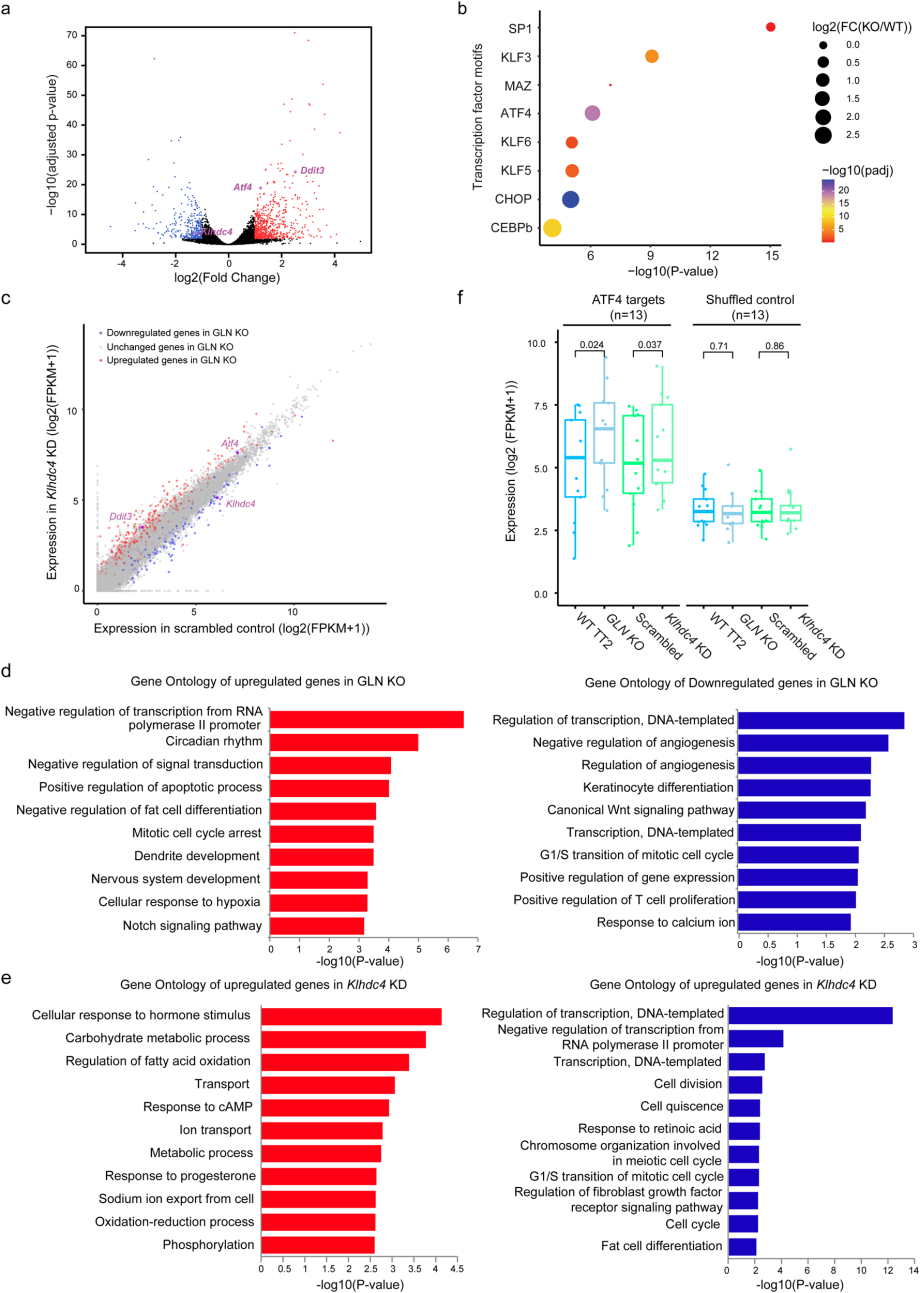
Polymorphic GLN regulates transcriptional network through ATF4. **a** Significantly dysregulated genes upon GLN KO are highlighted in the volcano plot. For each annotated gene, the negative log10-transformed two-tailed adjusted *P* values (*p*adj) from multiple testing using the Benjamini and Hochberg method are plotted against the log2-transformed fold change of FPKM (GLN KO/WT TT2). Fold change > 2 in either direction and *p*adj < 0.01 are used as the threshold to define dysregulated genes. Downregulated (*n* = 310) and upregulated genes (*n* = 647) upon GLN KO are labeled with blue and red, respectively. *Klhdc4*, *Atf4*, and *Ddit3* are highlighted in purple. **b** Bubble plot shows the most significantly enriched transcription factor binding motifs among the promoters (±1 kb of transcription start sites) of upregulated genes upon GLN deletion. The *x-*axis indicates negative log10-transformed one-tailed *P* values calculated by HOMER. The size of each bubble encodes negative log2-transformed fold change of expression in GLN KO cells compared to WT TT2. The color represents the negative log10-transformed two-tailed *p*adj of the expression change for each transcription factor. **c** Scatter plot shows the gene expression levels (log2(FPKM + 1)) in *Klhdc4* KD cells and scrambled control. The downregulated and upregulated genes upon GLN KO, as described in **a** are highlighted in blue and red, respectively. *Klhdc4*, *Atf4*, and *Ddit3* are highlighted in purple. **d**, **e** The most significantly enriched “Biological Process” Gene Ontology (GO) terms among upregulated genes (left) and downregulated genes (right) upon GLN KO (**d**) and *Klhdc4* KD (**e**) are shown. The negative log10-transformed one-tailed *P* values obtained from Fisher’s Exact test for each term are plotted in the bar charts. **f** Boxplots show the significantly increased expression of ATF4 targets (*n* = 13) upon GLN KO and *Klhdc4* KD compared to shuffled control genes (*n* = 13). The expression levels are shown as log2(FPKM + 1). The expression level of shuffled control is derived from the average of 50 times of shuffling. The centers and bounds of boxes refer to the median and quartile of all data points, respectively. The minima and maxima of boxplots indicate Quartile 1 − 1.5 × Interquartile range and Quartile 3 + 1.5 × interquartile range, respectively. One-tailed paired *T*-test is used to measure significance.

Transcriptomic analysis uncovered 957 genes that were dysregulated in GLN KO cells (Fig. 5a). These genes were distributed throughout the genome and were not restricted to chromosome 8 (Fig. S5a). In fact, other than *Klhdc4* no other genes within 1 Mb of the polymorphic GLN were defined as differentially expressed. Hence, besides its *cis*-regulatory effect on *Klhdc4*, GLN deletion also led to substantial downstream transcriptional changes. Therefore, we aimed to further explore the networks impacted by GLN and *Klhdc4* depletion. Surprisingly, the biological function of KLHDC4 had not been well characterized and little was known regarding its regulation. Accordingly, we opted to take a non-targeted approach to investigate the perturbed regulatory network by performing TF motif analysis on the promoter regions of dysregulated genes. Binding motifs for Activating Transcription Factor 4 (ATF4; 19.94% of the upregulated promoters compared to 12.8% of background) and C/EBPHomologous Protein (CHOP; 14.99% of the upregulated promoters compared to 9.5% of background) were significantly enriched among promoters of upregulated genes in GLN KO cells (Fig. 5b). Interestingly, ATF4 was reported as one of the core effectors of the integrated stress response (IRS), especially endoplasmic reticulum (ER) stress^50^. Upon stimulation, it induced the expression of *Ddit3*^51^, which encoded the CHOP/DDIT3 protein. In concert with ATF4, CHOP regulated downstream genes involved in various biological processes relating to programmed cell death, cellular metabolic processes, and translation^52,53^. We noted that *Atf4* and *Ddit3* were significantly upregulated in GLN KO and, to a lesser extent, in *Klhdc4 KD* cells (Fig. 5a, c). Thus, we hypothesized that the deletion of the polymorphic GLN and the reduced expression of *Klhdc4 *led to *Atf4* upregulation and consequently dysregulation of its downstream targets.

To test this model, we first analyzed the Gene Ontology (GO) terms and Kyoto Encyclopedia of Genes and Genomes (KEGG) pathways associated with dysregulated genes in both GLN KO and *Klhdc4* KD cells. The majority of the upregulated GO terms related to documented roles of ATF4 (Fig. 5d, e). For instance, ATF4 was reported to participate in circadian rhythm, various metabolism processes, and response to ER stress and hypoxia^54–56^. Moreover, DDIT3 and its targets were involved in negative regulation of transcription, fat cell differentiation, metabolic processes, the apoptotic process, and mitotic cell cycle arrest (Fig. 5d, e)^57,58^. On the other hand, the GO terms for downregulated genes in both GLN KO and *Klhdc4* KD cells were related to transcriptional regulation, in line with the function of ATF4 in IRS (Fig. 5d, e)^52^. Consistent with GO analysis, KEGG pathways associated with upregulated genes include lysosome and metabolism pathways, which are regulated by ATF4 under stress (Fig. S5b, S5c)^55,59^. While different GO terms and KEGG pathways were found, potentially due to the contribution of other dysregulated genes, ATF4-related processes were associated with dysregulated genes in both conditions. These findings suggested a central role of ATF4 in the transcriptional network regulated by the polymorphic GLN and KLHDC4.

To further investigate the role of ATF4, we quantified the expression levels of known ATF4-target genes. Specifically, by integrating publicly available RNA-seq and ChIP-seq datasets^60^, we focused on genes that were both bound by and regulated by ATF4^60^. The expression of these ATF4 targets was significantly increased in both GLN KO and *Klhdc4* KD cells compared to corresponding controls (Fig. 5f). These data supported the idea that the transcriptomic impacts observed in GLN mutants and KLHDC4-depleted cells involved ATF4. It is worth noting that *Atf4* was not differentially expressed between WT J1 and TT2 mESCs (Fig. S5d), potentially because the ATF4-DDIT3 pathway functions in response to stress. It was previously reported that mouse strains showed different responses to ER stress^61^. Intriguingly, while both C57BL/6 and 129S1/SvJ mice showed activation of ATF4-DDIT3 pathway upon tunicamycin (TM)-induced ER stress, a more dramatic increase was observed in 129S1/SvJ animals^61^. Consistently, we found that relative to C57BL/6, 129S1/SvJ mice showed greater transcriptional change of ATF4 targets upon TM treatment (Fig. S5e). Importantly, *Klhdc4* expression was unaltered from ER stress in both strains (Fig. S5f), suggesting that it functions upstream and transcriptionally regulates *Atf4*. These results collectively revealed that the ATF4-DDIT3 pathway was induced by decreased *Klhdc4* expression and the phenomenon was likely associated with strain-specific responses to stress. Further studies are required to delineate the precise mechanism of how KLHDC4 affects *Atf4* expression.

### Genome-wide analysis identifies additional polymorphic ERVs associated with differential epigenomic and transcriptomic patterns

**Fig. 6:**
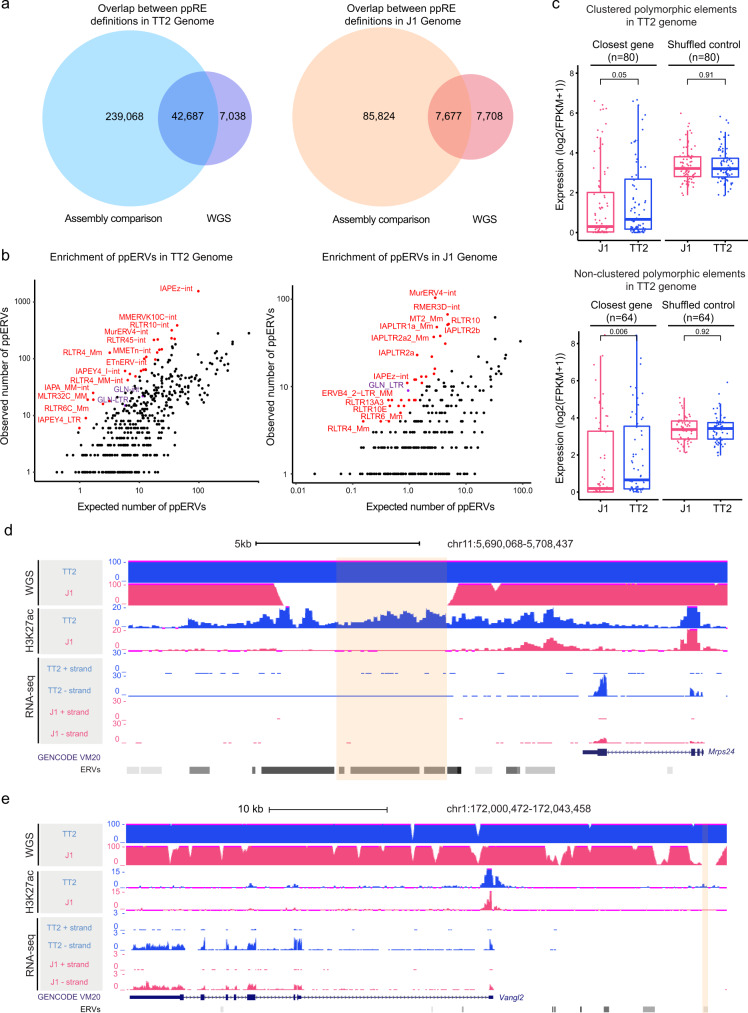
Genome-wide analysis identifies additional polymorphic ERVs capable of epigenomic and transcriptomic regulation. **a** Venn diagrams showing overlap between ppREs defined by comparing reference genome assemblies and by whole-genome sequencing (WGS) analysis in TT2 (left) and J1 (right). **b** Comparison of observed versus expected numbers among ppERVs subfamilies in TT2 (left) and J1 (right) show significant enrichment including IAP, ETn, RLTR4/MLV, ERVB4, and GLN elements. The subfamilies with observed/expected ratio > 7 in TT2 or observed/expected ratio > 10 in J1 and *P* value < 0.001 (in two-tailed hypergeometric distribution without multiple comparison adjustment) are labeled with red. GLN subfamilies are labeled with purple. **c** Boxplots indicate both clustered polymorphic elements and solo polymorphic elements in TT2 are associated with significantly higher expression of their closest genes in TT2 compared to J1 cells. The expression levels are shown as log2(FPKM + 1). The expression level of shuffled control is derived from the average of 50 times of shuffling. The centers and bounds of boxes refer to the median and quartile of all data points, respectively. The minima and maxima of boxplots indicate Quartile 1 − 1.5 × Interquartile range and Quartile 3 + 1.5 × interquartile range, respectively. One-tailed paired *T*-test was applied to calculate *P* values. **d** A genome browser screenshot illustrates an example of clustered polymorphic elements in the TT2 genome, which is associated with increased H3K27ac enrichment at its surrounding regions and upregulation of the nearby *Mrps24* gene. WGS datasets are shown as piled-up uniquely mapped reads. **e** A genome browser screenshot illustrates an example of solo polymorphic elements in the TT2 genome, which is associated with increased H3K27ac enrichment at its surrounding regions and upregulation of the nearby *Vangl2* gene.

Having demonstrated the capacity of the polymorphic GLN element to regulate nearby gene expression, which influenced strain-specific transcriptomic features, we expanded our analysis to a genome-wide scale. We attempted to identify potential polymorphic repetitive elements (ppREs) and potential polymorphic ERVs (ppERVs) between TT2 and J1 genomes. By comparing publicly available reference assemblies (mm10 and 129S1/SvJ), we identified 281,755 and 93,501 ppREs in C57BL/6 and 129S1/SvJ genomes, respectively. It is important to note that while these reference assemblies were reliable in uniquely aligned sequences, they varied in completeness and accuracy, especially for repetitive elements. For instance, the 129S1/SvJ assembly contained substantially more ambiguous nucleotides (N’s) than mm10. The high number of ppREs likely contained a proportion of false positives that arose from the different resolutions of the references. Moreover, structural variations and mutations between strains could also contribute to the overestimation of ppREs. Importantly, in this study, comparing the publicly available assemblies was insufficient to reflect the differences between our cell lines, since J1 mESCs were derived from 129S4/SvJae and TT2 cells were hybrids from C57BL/6 and CBA/J. To this end, we carried out whole-genome sequencing (WGS) of the TT2 and J1 genomic DNA (~130× coverage) to further refine and validate ppRE definitions. In total, 49,725 and 15,385 ppREs were defined by WGS in TT2 and J1, respectively. Subsequently, we overlapped ppREs defined by the two approaches, which yielded 42,687 elements in TT2 and 7,677 elements in J1 for downstream analysis (Fig. 6a).

Among these elements, we observed a significant overrepresentation of ppERVs (TT2: *P* < 1 × 10^−22^; J1: *P* < 1 × 10^−22^, from hypergeometric distribution), which constituted 30.48% (*n* = 13,011) and 22.81% (*n* = 1,751) of all ppREs in TT2 and J1 cells, respectively (Fig. S6a). In terms of ERV families, ERV-K elements were the most enriched among ppERVs (Fig. S6b), which was consistent with previous reports of ERV-K elements, such as IAP and ETn/MusD, showing high retrotransposition activity in murine genomes. Indeed, several subfamilies, including IAP and ETn/MusD, were enriched among ppERVs in TT2 and J1 cells (Fig. 6b). Moreover, other subfamilies with known instances of polymorphic insertions, including MLV (RLTR4) and ERVB4^34–37^, were similarly enriched in our analysis. As expected, the GLN subfamily was also enriched in ppERVs. In total, 37 and 10 GLN elements, including LTR and internal regions, were defined as polymorphic in TT2 and J1, respectively (Fig. 6b and Supplementary Data 3).

Given that the selection of new integration sites possesses inherent biases, repetitive elements tend to form clusters throughout the genome. Defining clusters as regions containing two or more ppREs with gap sizes of less than 20 bp, we found 61.8% and 58% of ERVs were within repeat element clusters in C57BL/6 and 129S1/SvJ genomes, respectively. In analyzing our polymorphic definitions, we observed both clustered and stand-alone ppERVs. To avoid confounding effects of multiple elements, we divided ppREs into clustered polymorphic elements and solo polymorphic elements in further analyses. In TT2 and J1 genomes, 68.3% and 41.7% of ppERVs were within ppRE clusters, respectively. In contrast, 31.7% and 58.3% (TT2: *n* = 4,129 J1: *n* = 1,021) ppERVs were solo elements. To ask whether ppERVs had regulatory functions, we defined epigenetically active ppERVs with H3K27ac enrichment (RPKM > 1 and length > 50 bp). In total, 524 and 125 ppERVs were identified as active in TT2 and J1, respectively. Among them, the majority were located in ppRE clusters (number of clusters in TT2: *n* = 197; J1: *n* = 27), with the remaining defined as solo ppERVs (TT2: *n* = 141; J1: *n* = 54). Taken together, 200 and 44 active ppERVs overlapped with the differential H3K27ac peaks (FC > 4, FDR < 0.001) defined between TT2 and J1 cells (TT2 peaks: *n* = 2,709; J1 peaks: *n* = 4,994), respectively. Strikingly, high H3K27ac enrichment was detected at the regions adjacent to active polymorphic clusters or solo elements (Fig. S6c, S6d). The genes closest to polymorphic clusters in TT2 had significantly higher expression in TT2 compared to J1 mESCs  (Fig. 6c). For instance, a TT2-defined ppREs cluster, composed of ERVs and SINEs, was differentially enriched with H3K27ac in TT2 cells (Fig. 6d). Concomitantly, the downstream *Mrps24* gene also had significantly higher expression in TT2, correlating with the differential proximal H3K27ac signal (Fig. 6d and Fig. S6g). While polymorphic clusters in J1 showed a similar trend of higher expression levels in J1 cells, no statistically significant difference was detected, which may be due to the low number of defined clusters (Fig. S6e). Nevertheless, solo ppERVs were also accompanied by increased expression of its closest genes (Fig. 6c and Fig. S6f). For example, an active polymorphic ORR1B1 element was defined in TT2, near to the promoter of *Vangl2*. Concordantly, higher *Vangl2* expression was detected in TT2 cells (Fig. 6e and Fig. S6g). Taken together, these data revealed that a proportion of the defined active ppERVs could affect the epigenetic and transcriptional states of proximal genomic loci.

## Discussion

Although neglected from most genomics studies, mounting evidence has demonstrated the capability of ERVs in regulating transcriptional networks^7–9^. Most notably, a subset of elements has been exapted to function in vital physiological processes. For instance, the ERV-derived placenta-specific Syncytin genes, which mediate syncytiotrophoblast fusion, are found in various placental mammals^11,62,63^. In fact, ERVs have been proposed to facilitate the evolution of mammalian placenta^64^. In different species, Syncytin genes are independently derived from species-specific ERVs, suggesting a functional but non-sequential conservation by convergent evolution^65^. Moreover, ERVs are involved in chromosomal rearrangements between *Mus musculus* and *Mus Pahari*^66^. On the other hand, transcripts initiated from lineage-specific ERVs in rat, mouse, and human oocytes are associated with divergent DNA methylation of nearby promoter CpG islands, implicating ERVs in driving inter-species divergence^67^. Similarly, polymorphic ERVs may also play a role in shaping intra-species divergence. Notably, transcripts derived from polymorphic ERVs are responsible for differential promoter DNA methylation among distinct mouse strains^67^. However, the scale and implications of this phenomenon remain unclear. Here, our study demonstrates that polymorphic ERVs, in part, provide the mechanisms that underlie intra-species transcriptomic variability. Interestingly, while the polymorphic GLN element’s transcript is only a minor contributor to the difference between WT C57BL/6 and 129 mESCs, its downstream impacts, including dysregulation of *Klhdc4* and *Atf4*, appear to be more substantial. Our data suggest that the element contributes to the differential stress responses of distinct mouse strains, giving rise to intra-species variance.

ERVs can influence the host transcriptome by supplying a repertoire of *cis*-regulatory elements, generating functional lncRNAs, or altering the 3D nuclear organization^8,12,68^. Since LTRs possess innate viral regulator sequences and TF-binding sites, many ERVs can exert promoter or enhancer effects on the neighboring loci when derepressed^36,69,70^. Our results show that the polymorphic GLN-LTR serves as both a promoter for the proviral transcript and an enhancer for nearby genes. To delineate the functions of the transcript and the LTR, we conducted deletion, knockdown, and epigenetic silencing experiments. When the GLN transcript was depleted by shRNA, we detected no global change to the transcriptome. On the other hand, knockout of the element and repression by CRISPRi yielded significant and consistent alterations. Therefore, we conclude that the polymorphic GLN functions predominately as a *cis*-regulatory element. Remarkably, of all full-length GLNs in the mouse genome, only this polymorphic element is active. Given that other GLN LTRs are virtually identical, the DNA sequence alone would not be sufficient to explain its unique properties. We infer that the genomic context of the integration is critical in determining the polymorphic element’s epigenetic and transcriptional states. Our results from knocking-in GLN via PiggyBac transposons demonstrated that only a subset of insertions display *cis*-regulatory signatures. This indicates that while the GLN sequence is capable of recruiting activators, the surrounding chromatin neighborhood determines its activity. In the case of this polymorphic GLN element in TT2 cells, it appears to have integrated into a prime location where it avoids DNA methylation- and H3K9me3-mediated silencing and thus can operate as an enhancer. Interestingly, initial reduction of *Klhdc4* expression and subsequent rescue over several rounds of cell division suggests the presence of redundant enhancers. Indeed, we found several differential H3K27ac peaks with higher signal in WT J1 and GLN KO cells as compared to WT TT2 cells (Fig. S4c). These results are analogous to the Temp enhancers or shadow enhancers identified in previous studies^45–48^. The deletion of specific enhancers is coupled with reduction of the target gene’s expression. However, over time, transcription was restored to WT levels through alternative enhancers^47^. In higher eukaryotic genomes, multiple enhancers can regulate an individual gene, which in part prevents sequence variations to severely impact gene expression. In the mouse genome, many genes have five or more redundant enhancers^49^. The deletion of individual elements results in no change, while deletion of enhancer pairs yielded substantial developmental phenotypes. Our results support the model that *Klhdc4* is regulated by multiple enhancers, including one provided by the polymorphic GLN element.

In the transcriptomic network regulated by the polymorphic GLN element, we found that *Klhdc4* plays a role in upregulating *Atf4* expression. Although the function of KLHDC4 remains largely elusive, our results revealed that decreased *Klhdc4* expression triggers the *Atf4* pathway, leading to dysregulation of genes in both apoptosis and adaptive responses to stress. In fact, relative to 129S1/SvJ, C57BL/6J mice were reported to be more susceptible to oxidative hepatic injury after acute arsenic exposure^71^, indicating different stress responses among mouse strains. It is worth noting that increased translation of *Atf4* mRNA is stimulated by phosphorylation of several eIF2 kinases in response to different cellular stresses. For instance, amino acid deprivation induces phosphorylation of GCN2, whereas increased PERK phosphorylation is induced by ER stress^50,72^. Regardless, both mechanisms lead to increased ATF4 protein levels. Our analysis shows that *Klhdc4* is not upregulated by ER stress and together with the increased expression of ATF4 targets in both the GLN KO and *Klhdc4* KD cells, we surmise that *Klhdc4* functions upstream of *Atf4*. Our findings also suggest that KLHDC4 regulates *Atf4* at the transcriptional rather than the translational level. These observations are consistent with a recent study demonstrating that deletion of *Slc7a5*, a WNT target gene, results in *Klhdc4* downregulation and activation of the ATF4 pathway^73^. Interestingly, in addition to ATF4, TF-binding motifs of KLF proteins are also enriched at promoters of dysregulated genes in GLN KO cells. It is worth noting that *Klf3* and *Klf5* genes are also mildly upregulated in GLN KO cells (Fig. 5b). Previous studies have reported that *Klf4* and *Klf5* are regulated by ATF4 and its downstream targets^74,75^. Hence, transcriptional changes observed in our datasets may result in part from ATF4’s control of KLF proteins.

Remarkably, we identified thousands of ppERVs in both mESC lines, revealing that polymorphic ERVs across mouse genomes may be more prevalent than previously appreciated. Of note, the number of ppERVs in TT2 mESCs is approximately 5–6-fold more than that in J1, which may be due to the difference in the integrity of the genome assemblies. In fact, although two assemblies are of similar length and GC content, mm10 assembly contains 2,862 N’s per 100 kb, while the 129S1/SvJ assembly contains 14,858 N’s per 100 kb. This difference could contribute to false definitions of polymorphic elements. In addition, the presence of a small number of highly active elements in the either background may lead to different numbers and composition of ppREs. For instance, IAPs are highly polymorphic in the C3H strains, which were suggested to originate from a “master copy” of the ERV^24,29^. Indeed, the top enriched ppERV subfamilies are different between TT2 and J1 cells, which could arise from distinct master copies. Importantly, we discovered that active ppERVs are associated with increased H3K27ac signal and gene expression in adjacent sequences. Given the observations of the polymorphic GLN and pervasiveness of ppERVs, it will be necessary to further analyze the functions of other polymorphic elements. In addition, *de novo* genome assembly with a focus on annotating repetitive sequences will offer additional details to our results.

Our findings elucidated the function of the polymorphic GLN element in transcriptomic regulation via fine-tuning *Klhdc4* expression. Consequently, this role contributes to intra-specific variation among commonly used mouse strains. Moreover, the scope of active polymorphic ERVs and their impact on the epigenome may be much larger than previously expected. While the ERVs are mostly not conserved in sequence between the mouse and human genomes, the mechanism of how they influence host genes can be comparable. For instance, polymorphic HERV-K HML-2 elements were reported to be associated with several human pathologies. The polymorphic insertion of the HERV-K113 element, a full-length HERV-K HML-2 provirus, had a significantly higher prevalence in the genomes of specific patients of Sjögren’s syndrome, multiple sclerosis, systemic lupus erythematosus, and rheumatoid arthritis^70,76^. Moreover, a polymorphic HERV-K HML-2 LTR was discovered in an intron of *RASGRF2*, a gene associated with dopaminergic activity and addiction. The insertion was suggested to alter *RASGRF2* expression and was more frequently detected in intravenous drug users^69^. Indeed, a global analysis suggested that polymorphic HERV-K elements were statistically associated with diverse expression quantitative trait loci (eQTLs) and complex human diseases, including neurological and immunological diseases^22^. Given the clinical implications, it would be compelling to investigate whether an analogous mechanism underlies the polymorphic HERVs and their reported associations with individual-specific pathologies and phenotypes.

## Methods

### Cell culture

Mouse embryonic stem cells (mESCs) were cultured in mESC completed medium (Dulbecco’s modified Eagle’s medium (DMEM) supplemented with 15% fetal bovine serum (Gibco), 1% Glutamine (Gibco), and 1% penicillin–streptomycin (Gibco), 1% MEM non-essential amino acids (Gibco), 1% sodium pyruvate (Gibco), 2% HEPES(Gibco), 0.01% mouse LIF (Sigma), 0.5% 2-mercaptoethanol (Sigma)). Medium was changed every 48 h and passaged when cells reach 80% confluency.

Human embryonic kidney 293 (HEK293) cells were maintained in DMEM supplemented with 10% fetal bovine serum (Gibco), 1% glutamine (Gibco), and 1% penicillin–streptomycin (Gibco). Medium was changed every 48 h and passaged when cells reached 80% confluency.

### Chromatin immunoprecipitation (ChIP) sequencing and qPCR

Micro-ChIP was performed as described previously^77^. Briefly, 5 × 10^5^ crosslinked cells were resuspended in lysis buffer and sonicated with the Covaris S220 sonicator continuously for 400 s at 175 W. Fragmented chromatin was added to Protein A Dynabeads (Thermo Fisher Scientific) that were bound beforehand to antibodies of interest and incubated at 4 °C for 40 h. Captured chromatin was washed four times with RIPA buffer and subsequently eluted at 37 °C for 1 h in elution buffer. Reverse crosslinking was done by incubation at 68 °C for 4 h with Proteinase K. Eluted DNA was purified with the QIAquick PCR Purification Kit (Qiagen). Purified DNA was then quantified with Qubit 3.0 Fluorometer. For NGS, DNA libraries were prepared using the KAPA HyperPrep Kit (Roche) according to the manufacturer’s protocol. Size distribution of DNA fragments of final libraries were confirmed using an Agilent Fragment analyzer with DNF 474 kit (Agilent) and DNA libraries were quantified with a Qubit 3.0 Fluorometer (Thermo Fisher Scientific) and the KAPA library quantification kit (Roche). All the DNA libraries were pooled and normalized to 4.4 nM and then submitted for NGS.

Native ChIP was performed as described previously^78^. Briefly, 1 × 10^7^ cells were resuspended in douncing buffer and lysed. Subsequently, chromatin was digested with 450 U/ml Micrococcal nuclease (MNase) for 10 min at 37 °C. EDTA was added to quench the digestion. Nuclear membrane was lysed by incubating 1 h in hypotonic lysis buffer. Immunoprecipitation was performed by adding the digested chromatin into Dynabeads M-280 Sheep Anti-Mouse IgG (Thermo Fisher Scientific) or Dynabeads M-280 Sheep Anti-Rabbit IgG (Thermo Fisher Scientific) that were pre-bound to antibodies of interest. Precipitated chromatin was washed twice with wash buffer, once with final wash buffer, and eluted at 68 °C for 1 h in elution buffer together with ribonuclease (RNase). Eluted DNA was purified with QIAquick PCR Purification Kit (Qiagen) according to the manufacturer’s protocol. Purified DNA was quantified with NanoDrop 2000C (Thermo Scientific) and subjected to qPCR with LightCycler® 480 Instrument II (Roche). All the primers and antibodies used in this study are listed in Supplementary Data 4 and Table S2, respectively.

### Stranded total RNA sequencing (RNA-seq) and reverse transcription qPCR (RT-qPCR)

RNA was extracted from 1 × 10^7^ cells with the RNeasy Mini Kit (Qiagen) according to the manufacturer’s protocol and the concentration was measured with a Qubit 3.0 Fluorometer. For NGS, 1 µg of RNA was treated with the Ribo-off rRNA Depletion Kit (Human/Mouse/Rat) (Vazyme) according to the manufacturer’s protocol and the concentration was measured again. Subsequently, 10–100 ng of rRNA depleted RNA was used to prepare RNA-seq library with QIAseq Stranded RNA Library Kits (Qiagen). Size distribution of DNA fragments of final libraries were confirmed using an Agilent Fragment analyzer with the DNF 474 kit and libraries were quantified with a Qubit 3.0 Fluorometer and the KAPA library quantification kit. All the DNA libraries were pooled and sequenced on the Illumina NextSeq 500 platform.

For RT-qPCR, extracted RNA was subjected to DNase I (NEB) treatment and purified with Agencourt® RNAClean™ XP (Beckman Coulter) before first-strand synthesis. First-strand synthesis was carried out by using Superscript III Reverse Transcription System (Thermo Scientific) according to the manufacturer’s protocol. cDNA was then analyzed by qPCR on LightCycler® 480 Instrument II. Primers used in this study are listed in Supplementary Data 4.

### Western blot

Western blot was performed as previously described^79^ with minor modifications. Briefly, 1 × 10^7^ cells were lysed in the lysis buffer. Whole-cell lysates were fractionated by SDS-PAGE and the proteins were transferred to a PVDF membrane using a liquid transfer system (Bio-Rad). The membrane was blocked in 10% non-fat milk in TBST (1 × TBS with 0.1% Tween-20) for 1 h at room temperature followed by incubation with a primary antibody in 5% BSA (NEB) at 4 °C overnight with continuously shaking. After repeated washing, the membrane was then incubated in 10% non-fat milk containing a horseradish peroxidase conjugated secondary antibody for 1 h and washed in 1× TBST. The resultant bands were visualized by Clarity™ Western ECL Substrate (Bio-Rad). Primary antibodies and the dilution factors used are listed in Table S2.

### Molecular cloning

For generating KO, KD, and CRISPRi targeting constructs, complementary pairs of oligos were annealed at 95 °C for 5 min followed by 25 °C for 1 h to generate double stranded insert. All oligos used are listed in Supplementary Data 4. Vectors were digested with corresponding enzymes and purified by the MinElute® Gel extraction kit (Qiagen). All the enzymes and vectors used in this study are listed in Table S3. A 1:3 ratio of digested vector to oligos was ligated with T4 DNA ligase (NEB) at 4 °C overnight. For knockout experiments, PX330A-1×2 vector with GFP reporter and PX330S-2 vector were used to construct sgRNA-containing CRISPR-cas9 constructs. For knockdown experiments, pSIH1-H1-copGFP shRNA expression lentivector was used. For CRISPRi experiments, pL-CRISPR.EFS.GFP was used to carry sgRNA and was transfected together with Lenti-dCas9-KRAB-blast vector. For PiggyBac experiments, previously described PB513Re vector was used to construct GLN-containing vectors^68^. The intact GLN sequence was amplified with primers listed in Supplementary Data 4. For luciferase assay, pGL3 vectors (basic vector, enhancer vector, and promoter vectors) (Promega) were used. The sequences of GLN-LTR and GFP were amplified with primers listed in Supplementary Data 4.

### Generation of GLN KO cell lines

TT2 cells were seeded at 1.5 × 10^5^ cells per well of six-wells plate well, 1 day prior to transfection. mESCs were transfected with 5,000 ng of PX330A-GFP vector containing two sgRNAs with Lipofectamine® 3000 (Thermo Fisher Scientific) according to the manufacturer’s protocol. Cells were incubated overnight at 37 °C in 5% CO_2_ and medium was changed after 18 h. Three days post-transduction, FACS analysis was performed. Single-GFP-positive cells were sorted into individual 96-wells plate wells with 0.1 ml mESC completed medium. The sorted cells were allowed to grow for 2 weeks and monitored daily by microscopy, until single colonies were obtained. To confirm the deletion of GLN elements, genotyping was performed with specific primers (Supplementary Data 4).

### Generation of GLN knockdown and GLN-CRISPRi cell lines

HEK293 cells were seeded at 1.5 × 10^6^ cells per well of six-wells plate well and transfected with lentiviral constructs, pMD2.G (containing VSV-G envelope) plasmids, and pCMVR 8.74 packaging vector with Lipofectamine® 3000 (Thermo Fisher Scientific) according to the manufacturer’s protocol. Cells were incubated overnight at 37 °C in 5% CO_2_ and media was changed after 18 h. Viral supernatant were harvested at day 2 and 3 post-transfection. mESCs were transduced with the supernatant with 8 µg/ml filter-sterilized polybrene. FACS analysis was performed on cells transduced with shRNA-containing vectors on day 3 post-transduction. GFP-positive cells were then sorted by FACS using BD FACSAria™ III (BD Biosciences).

For CRISPRi experiments, blasticidin selection (10 μg/ml) was carried out after transduced mESCs reached confluence. After selection, the remaining transduced cells were subjected to FACS to obtain GFP-positive population for future experiments.

### Luciferase assay

Dual-Luciferase® Reporter Assay System (Promega E1960) was used to perform luciferase assay for GLN-LTR. Briefly, mESCs were seed at 1.5 × 10^5^ per well of six-wells plate well 1 day prior to transfection. The cells were transfected with 1,000 pmol of pGL3 vectors containing either GLN-LTR sequence or GFP sequence together with 20 pmol of *Renilla* vector with Lipofectamine® 3000. Cells were incubated overnight at 37 °C in 5% CO_2_ and media was changed after 18 h. Once the cells reach confluence, 500 μl of 1× Passive Lysis Buffer was added into the cells. The plates were rotated at room temperature for 15 min cell lysis. Subsequently, 1 ml of lysate was mixed with 100 μl LAR II and 100 μl Stop & Glo® Reagent. Luciferase activity was measured with Spectronic Genesys 5 UV/Visible Spectrophotometer (ALT) after each addition to record firefly luciferase and *Renilla* luciferase, respectively.

### Generation of GLN-PiggyBac (GLN-PB) cell lines

GLN-PB clones were generated as previously described^68^ with minor modifications. Briefly, 1 day prior to transfection, 1.5 × 10^5^ J1 cells were seeded in six-well plates. Five micrograms GLN-PB513Re plasmid and 0.5 μg transposase were transfected into the cells using Lipofectamine Stem Transfection Reagent (Thermo Fisher Scientific) according to the manufacturer’s protocol. Puromycin selection (3 μg/ml) was done and single-cell clones were obtained by serial dilution.

### Inverse PCR

Inverse PCR was conducted as previously described^80^ with minor modifications. Briefly, genomic DNA of J1 and PB clones was isolated with Bradley’s protocol. Subsequently, 1 μg of genomic DNA was digested with *Mbo*I (NEB) and purified with QIAquick PCR Purification Kit. Two hundred and fifty nanograms of digested DNA was circularized with T4 ligase (NEB) at 16 °C for overnight. PCR was performed with specific primers and the amplicons were purified with 0.8× of Ampure XP beads. Finally, the purified PCR products were cloned into PCR2.1 vectors (Invitrogen) and analyzed by Sanger Sequencing. Of note, PB14 had at least one insertion within an IAP element that rendered its precise location unclear. Thus, the element was excluded and subsequent analysis focused only on remaining validated integrations.

### Whole-genome sequencing

Genomic DNA of TT2 and J1 cells was isolated with Bradley’s protocol as described previously^81^. Briefly, 1 × 10^7^ cells were resuspended in 200 µl of TE buffer and 200 µl of 2× Bradley’s reagent was added with 0.1 mg of Proteinase K (Invitrogen). The solution was then incubated overnight at 55 °C for lysis. Eight hundred microliters ice cold 100% EtOH with 75 mM sodium acetate were slowly added. After incubation at room temperature for 1 h, DNA was pelleted by centrifugation and washed twice with 70% EtOH. Finally, DNA pellets were resuspended in 40 µl of TE buffer. The size distribution of DNA was determined by 1.5% agarose gel and the concentration was determined by a Qubit 3.0 Fluorometer. The purified DNA was fragmented by sonication and subjected to NGS on the MGISEQ-2000-RS platform (BGI Genomics).

### Bioinformatics analysis

#### Publicly available assemblies and datasets used in this study

In this study, we aligned sequencing data to publicly available mm10 and 129S1/SvJ mouse reference genome, separately. mm10 and 129S1/SvJ genomes were downloaded from UCSC and NCBI, respectively (GCA_001624185.1). The CBA/J assembly was downloaded from NCBI (GCA 001624475.1). These assemblies were used for analyzing the presence of the polymorphic GLN element. Basic information of our datasets is listed in Table S4. Published RNA-seq and ChIP-seq datasets were downloaded from GEO under the following accession numbers:

**Table:** 

Cell type/tissue	Type	Accession number	Citation
Liver from 129S1/SvJ and C57BL/6 mice	RNA-seq	GSE45684	^44^
Various tissues from C57BL/6 mice	RNA-seq	GSE29184	^82^
TT2 mESCs	H3K9me3 ChIP-seq	GSE47887	^83^
129S1/SvJ and C57BL/6 mice treated with DMSO or tunicamycin	RNA-seq	GSE63756	^61^

### ChIP-seq data analysis

Paired-end sequencing reads were mapped to mm10 reference genome and 129S1/SvJ genome using Bowtie v2.3.5.1 aligner^84^ with the parameters -q -N 1 -L 25 -X 500 —no-discordant —no-mixed. The output SAM files were converted into BAM format and sorted according to genomic coordinate using SAMtools^85^, followed by PCR duplicates removal with Picard tool 2.3.0. The resulting BAM file was converted into BED format with BEDTools^86^ and to WIG and BigWig files for visualization on the UCSC genome browser. For normalization, RPKM values were generated for 100 bp bins and Input-subtracted RPKM was calculated by subtracting RPKM values of input from RPKM of IP per bin.

Peak calling was performed by MACS2 (ref. ^87^) with the parameters –nomodel —keep-dup all -p 0.05. To identify differential peaks between two datasets, we first merged their peaks with BEDTools^86^. Subsequently, read counts of each merged peak were calculated and normalized by sequencing depth. Statistical analysis was conducted based on Poisson distribution as previously described^88^. Finally, the peaks with fold change above 4 and FDR below 0.001 were defined as differential peaks.

### RNA-seq analysis

Reads were aligned to mouse reference genome (mm10 and 129S1/SvJ) with STAR^89^ with the following parameters: —outFilterMultimapNmax 1 —alignSJoverhangMin 8 —alignSJDBoverhangMin 1 —outFilterMismatchNmax 999 —outFilterMismatchNoverReadLmax 0.04 —alignIntronMin 20 —alignIntronMax 1000000 —alignMatesGapMax 1000000 —outSAMstrandField intronMotif —quantMode TranscriptomeSAM —sjdbScore 1. Only uniquely mapped reads were included for further analysis. The output SAM files were converted into BAM format and sorted according to genomic coordinate using SAMtools^85^. Subsequently, the BAM files were converted into BedGraph and BigWig formats for visualization on the UCSC genome browser^89^. For normalization, sorted BAM files were used to calculate fragments per kilobase per million reads (FPKM) of genes with RSEM^90^ with the following parameters: —bam —estimate-rspd —calc-ci —seed 12345 —no-bam-output —ci-memory 70000 —paired-end. Differentially expressed genes were defined with DESeq2^91^. For ERVs, FPKM values were calculated per element as previously described^88^. Briefly, BAM files were converted into BED files by SAMtools^85^ with parameter –split followed by calculating RPKM values of each ERV element with the BED files.

### Generation of aggregation plots and heatmaps

BigWig files generated from RNA-seq and ChIP-seq datasets were subject to “computeMatrix” function of deepTools^92^ together with BED files documenting the target regions. Subsequently, plots were generated by “plotHeatmap” function with the parameter –perGroup.

### Maximum likelihood phylogeny tree with relative time

To generate maximum likelihood phylogeny tree, the coordinates of all intact GLN elements and DNA sequence were extracted from RepeatMasker definitions from UCSC for the mm10 mouse assembly. The full-length GLN consensus sequence was obtained from the Dfam database^93^. The sequences were used to generate maximum likelihood phylogeny tree by with the Molecular Evolutionary Genetics Analysis software (MEGA-X) with the following settings: bootstrap of 1,000, substitution model to general time reversible model, rates among sites to gamma distribution with invariant sites, and number of discrete gamma categories to 5 (ref. ^94^). Finally, DNA sequence and the resulting phylogeny tree were used to generate maximum likelihood phylogeny relative time tree with the “clock” function in MEGA-X, with the consensus sequence as the outgroup.

### Hierarchical clustering and PCA

FPKM values of genes calculated by RSEM were extracted and a threshold of FPKM > 0.1 in at least one sample was applied. Next, ANOVA test was applied to all the samples included in the analysis and the *P* value followed by *F* value was used to sort the genes and obtain the most variably expressed gens. Then the FPKM value of the top genes (*n* = 1,000 for 8 samples and *n* = 2,000 for 12 samples) were used to calculate the Pearson score of samples and R function “pheatmap” was utilized to generate the hierarchical clustering heatmap with (1−Pearson). Batch effect was removed by the “ComBat” function of R package “sva”^95^. The FPKM values of the top genes were analyzed by R packages “factoextra” with default parameters produce to PCA plot.

### Statistical analysis of overlapped differentially expressed genes

For each differentially expressed genes in TT2 and J1, as well as *Klhdc4* KD cells, number matched gene lists were generated by random selection. The selection was repeated 1,000 times. Subsequently, differentially expressed genes in GLN KO cells were compared with the randomly selected gene lists and the numbers of overlapped genes were used to generate the histograms/density curves, which represent random distributions. The one-tailed *P* value was generated via non-parametric bootstrapping.

### Gene Set Enrichment Analysis

FPKM values of all the genes in the compared datasets were extracted and analyzed by GSEA 3.0 (ref. ^96^) together with list of differentially expressed genes in corresponding datasets. The number of permutations was set to 1000 and permutation type was set to gene set.

### Enrichment and prediction of TF binding motifs

To identify transcription factor-binding motifs enriched at promoter regions of differentially expressed genes, coordinates ±1 kb of TSS of these genes were extracted from GTF file. Subsequently, findMotifsGenome.pl from HOMER was utilized to obtain the enriched motifs with background set as whole genome and size as given^97^.

For scanning of individual locus, DNA sequence of LTR region of the polymorphic GLN element was submitted to the TFBIND online tool (https://tfbind.hgc.jp/) with default setting^98^.

### GO and KEGG analysis

GO terms and KEGG pathways of differentially expressed genes were generated by DAVID (Database for Annotation, Visualization and Integrated Discovery)^99^. The top significant terms were shown in bar charts.

### WGS data analysis

WGS reads of TT2 and J1 cell lines were aligned to both mm10 (C57BL/6) and 129S1/SvJ assemblies separately. Bowtie v2.3.5.1 (ref. ^84^) was used to align paired-end reads to each reference with parameters: -N 1 -L 25 -X 600 —no-discordant —no-mixed. Only uniquely aligned reads (MAPQ > 30 and AS > XS) were included in downstream analysis. The output SAM files were then converted into BAM files and sorted according to genomic coordinate using SAMtools^85^. The resulting BAM files were subsequently converted into BED file by BEDTools^86^. The BED file was then converted into BedGraph and BigWig files for visualization on the UCSC genome browser.

### Identification of potential polymorphic elements

To identify ppREs with publicly available assemblies, each chromosome was separated into Fasta files for analysis. For ppREs specific in mm10 assembly, Fasta files of mm10 were input as the reference file and those of 129S1/SvJ were input as the query file using the “nucmer” function of MUMmer v4.0.0rc1 with the parameters: -l 20 -g 50 -c 200 (ref. ^100^). Next, the gaps were extracted and overlapped with RepeatMasker annotations by BEDTools with parameters -f 1 (ref. ^86^) and the resulting elements were defined as potentially polymorphic in mm10 assembly. For ppREs specific in 129S1/SvJ assembly, Fasta files of 129S1/SvJ were input as reference file and those of mm10 were input as query, followed by the same processing.

To identify ppREs and ppERVs with WGS data, overlap with RepeatMasker definitions were applied with BEDTools^86^. The elements with coverage >10 reads in 1 cell line and no coverage in the other cell line were defined as potentially polymorphic. For example, the elements in mm10 assembly that were covered by more than 10 reads in TT2 WGS but had no reads in the J1 library were defined as potential polymorphic elements in TT2 cell line. The final list of ppREs and ppERVs were defined by overlapping the elements from both methods and only retaining those that were mutually identified as polymorphic.

### Subfamily enrichment analysis of ppERVs

A custom approach was used to calculate observed and expected numbers of polymorphic elements of each subfamily. The expected numbers were estimated with (*n*/*N*)*X (*n* = total number of elements of each subfamily; *N* = total number of ERV elements in genome; *X* = total number of the defined ppERVs). *P* value of each subfamily was calculated by two-tailed hypergeometric distribution.

### Analysis of active ppERVs

To define active ppERVs, H3K27ac ChIP-seq datasets were aligned to both assemblies and RPKM of ppERVs were calculated. The ppERVs with RPKM > 1 and size > 50 bp were identified as active ppERVs in the corresponding mESC line. To obtain list of closest genes to active ppERVs, “closest” function of BEDTools^86^ was used to identify the genes with promoters closest to active ppERV locations. In downstream analysis, only genes with expression in at least one sample were considered.

### Assessment of publicly available assemblies

To define the number of ambiguous nucleotides and to assess the quality of the publicly available mm10 and 129S1/SvJ assemblies, we employed QUAST^101^ with non-reference mode and default parameters.

### Reporting summary

Further information on research design is available in the Nature Research Reporting Summary linked to this article.

## Supplementary information

Supplementary Information

Description of additional Supplementary File

Supplementary data 1

Supplementary data 2

Supplementary data 3

Supplementary data 4

Reporting Summary

## Data Availability

The data that support this study are available from the corresponding author upon reasonable request. All the sequencing data generated in this study have been deposited in the National Center for Biotechnology Information (NCBI) database under GEO accession code GSE165214. The consensus sequence of GLN used in this study was obtained from Dfam database (https://dfam.org/home). All previously published datasets used in our integrative analyses are found in the NCBI GEO database under the following accession codes: RNA-seq datasets of murine liver (GSE45684), RNA-seq datasets from C57BL/6 mouse tissues (GSE29184), RNA-seq datasets of 129S1/SvJ and C57BL/6 mice treated with DMSO and/or tunicamycin (GSE63756), and H3K9me3 ChIP-seq datasets from TT2 mESCs (GSE47887). Source data are provided with this paper.

## Source data

Source data
