# Supplementary material for: Mouse strain-specific polymorphic provirus functions as cis-regulatory element leading to epigenomic and transcriptomic variations
Source: Nat Commun. 2021 Nov 9;12:6462. doi: 10.1038/s41467-021-26630-z (PMC8578388; doi:10.1038/s41467-021-26630-z)
Supplement: Supplementary file 1 — Supplementary Information [file 41467_2021_26630_MOESM1_ESM.pdf]

Supplemental Information:

Mouse strain-specific polymorphic provirus functions as *cis*-regulatory element leading to epigenomic and transcriptomic variations

Xuemeng Zhou, Tsz Wing Sam, Ah Young Lee and Danny Leung

Supplemental Figures, supplemental tables, and supplemental references.

Figure S1

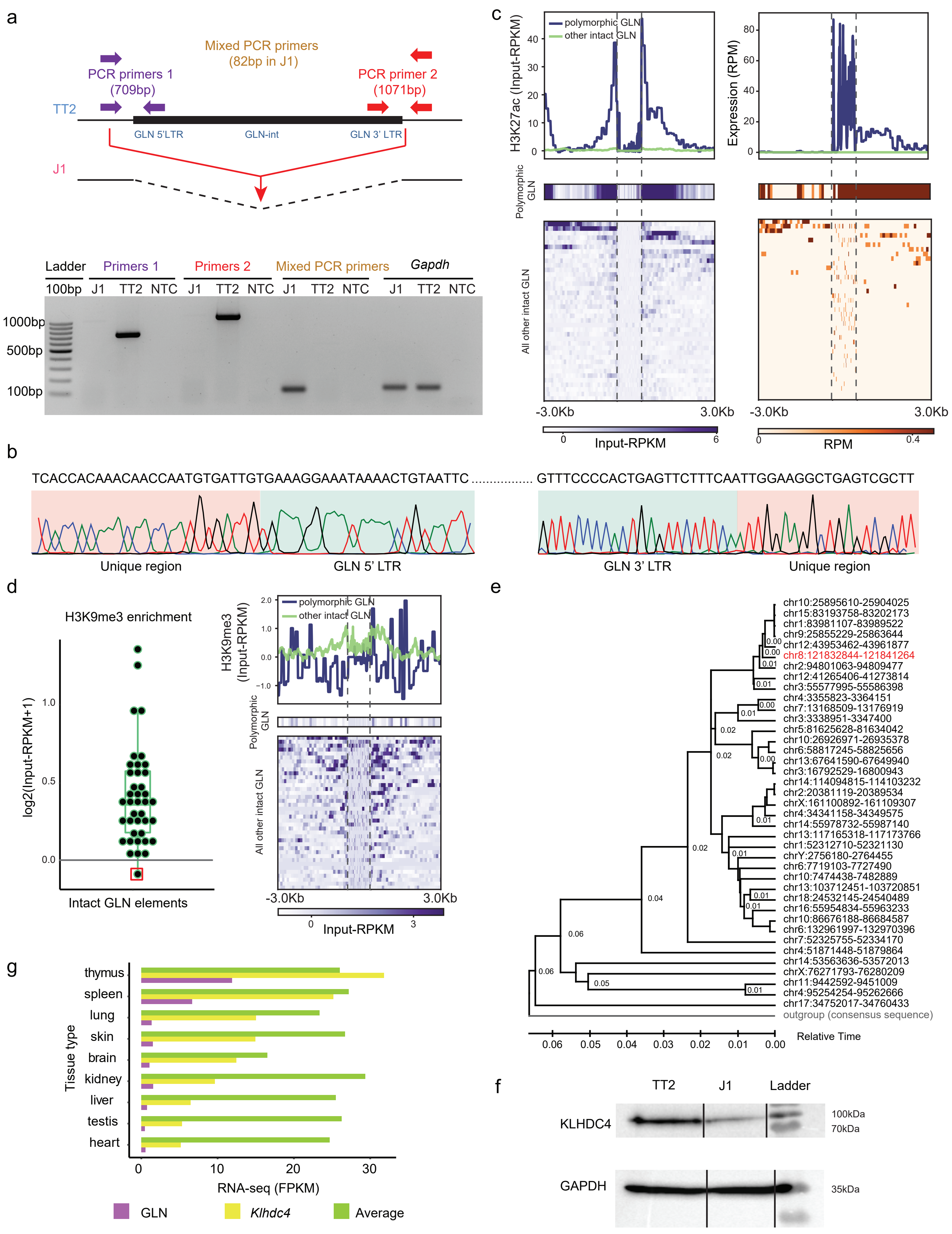
a
Mixed PCR primers
(82bp in J1)
PCR primers 1
(709bp)
PCR primer 2
(1071bp)
TT2
GLN 5'LTR
GLN-int
GLN 3' LTR
J1
Ladder
Primers 1
Primers 2
Mixed PCR primers
Gapdh
100bp
J1
TT2
NTC
1000bp
500bp
100bp
b
TCACCACAAACAACCAATGTGATTGTGAAAGGAAATAAAACTGTAATTC .... GTTTCCCCACTGAGTTCTTTCAATTGGAAGGCTGAGTCGCTT
Unique region
GLN 5' LTR
GLN 3' LTR
Unique region
c
polymorphic GLN
other intact GLN
H3K27ac (Input-RPKM)
Expression (RPM)
Polymorphic GLN
All other intact GLN
-3.0Kb
3.0Kb
Input-RPKM
RPM
d
H3K9me3 enrichment
log2(Input-RPKM+1)
Intact GLN elements
H3K9me3 (Input-RPKM)
-3.0Kb
3.0Kb
Input-RPKM
e
chr10:25895610-25904025
chr15:83193758-83202173
chr1:83981107-83989522
chr9:25855229-25863644
chr12:43953462-43961877
chr8:121832844-121841264
chr2:94801063-94809477
chr12:41265406-41273814
chr3:55577995-55586398
chr4:3355823-3364151
chr7:13168509-13176919
chr3:3338951-3347400
chr5:81625628-81634042
chr10:26926971-26935378
chr6:58817245-58825656
chr13:67641590-67649940
chr3:16792529-16800943
chr14:114094815-114103232
chr2:20381119-20389534
chrX:161100892-161109307
chr4:34341158-34349575
chr14:55978732-55987140
chr13:117165318-117173766
chr1:52312710-52321130
chrY:2756180-2764455
chr6:7719103-7727490
chr10:7474438-7482889
chr13:103712451-103720851
chr18:24532145-24540489
chr16:55954834-55963233
chr10:86676188-86684587
chr6:132961997-132970396
chr7:52325755-52334170
chr4:51871448-51879864
chr14:53563636-53572013
chrX:76271793-76280209
chr11:9442592-9451009
chr4:95254254-95262666
chr17:34752017-34760433
outgroup (consensus sequence)
Relative Time
f
TT2
J1
Ladder
KLHDC4
GAPDH
100kDa
70kDa
35kDa
g
thymus
spleen
lung
skin
brain
kidney
liver
testis
heart
Tissue type
RNA-seq (FPKM)
GLN
Klhdc4
Average

Figure S1: Validation and characterization of polymorphic GLN integration and its association with the differential gene expression

a) PCR confirms the presence of the polymorphic GLN element in TT2 and not J1 genomes. Design of the primers is shown on the top schematic diagram. *Gapdh* primer serves as positive control. Experiments were repeated with two biological replicates (n=2). **b)** Sanger sequencing result validates the presence of the polymorphic GLN element in TT2 genome. **c)** Aggregation plots and heatmaps show the polymorphic GLN element displays higher H3K27ac signal (Input-RPKM) (left) and expression level (RPM) (right) compared to other intact GLN elements. **d)** Boxplot of the H3K9me3 ChIP-seq signal (log2(Input-RPKM+1)) (left), aggregation plot and heatmaps of the H3K9me3 ChIP-seq signal (Input-RPKM) (right) on all the intact GLN elements (n=40) in the mm10 reference assembly show the polymorphic GLN element is the only intact GLN element not marked by H3K9me3. Grey line in the boxplot denotes log2(RPKM+1) value of zero. The polymorphic GLN element is marked by a red box in boxplot. The center and bounds of boxes refer to the median and quartile of all the dots, respectively. The minima and maxima of boxplots indicate Quartile 1 - 1.5 x Interquartile range and Quartile 3 + 1.5 x Interquartile range, respectively. Notably, the low RPKM of H3K9me3 compared to H3K27ac is in part due to the short read length (36bp) of publicly available dataset. **e)** Phylogenetic analysis shows the polymorphic GLN element (red) is relatively young compared to other intact GLN elements. The time tree is generated by applying the RelTime method [1,2] to the phylogenetic tree. Branch lengths are calculated using the Maximum Likelihood (ML) method and the General Time Reversible substitution model [3]. All divergence times shown are relative time measurements. The estimated log likelihood value of the tree is -305733.12. A discrete Gamma distribution was used to model evolutionary rate differences among sites (5 categories (+G, parameter = 7.0071)). The rate variation model allowed for some sites to be evolutionarily invariable ([+I], 0.09% sites). Evolutionary analyses were conducted in MEGA X [4]. **f)** Western blot of KLHDC4 in TT2 and J1 cell lines reveals higher protein level in TT2 compared to J1. GAPDH is used as loading control. Western blots were repeated with independently derived lysates (n=2). **g)** Bar chart shows expression level (FPKM) of *Klhdc4*, polymorphic GLN, and the average expression level of all genes in distinct tissues of C57BL/6 strain. Average expression level of all genes (green bar) serves as control.

Figure S2

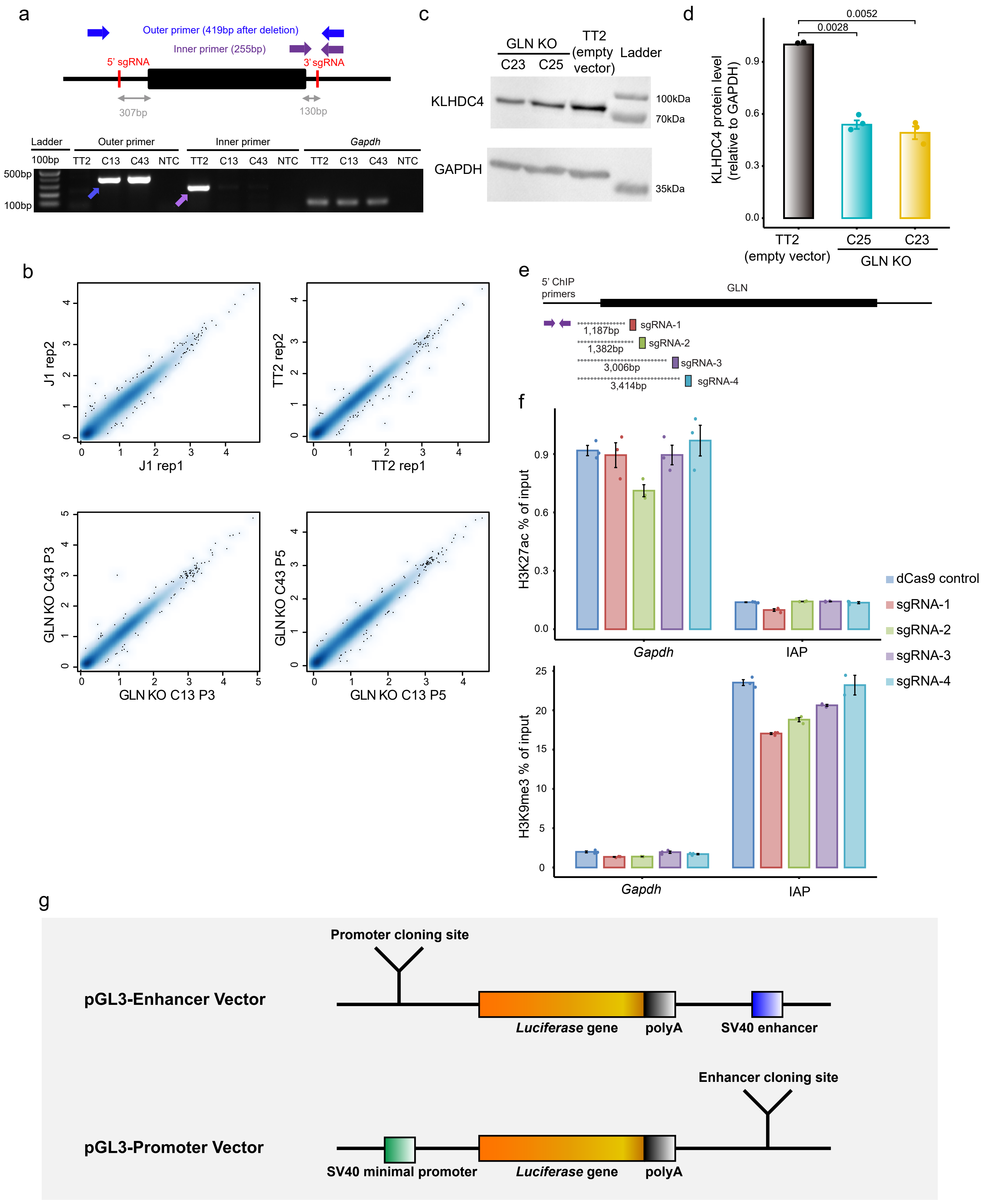

a
Outer primer (419bp after deletion)
Inner primer (255bp)
5' sgRNA
3' sgRNA
307bp
130bp
Ladder
Outer primer
Inner primer
Gapdh
100bp
TT2 C13 C43 NTC TT2 C13 C43 NTC TT2 C13 C43 NTC
500bp
100bp
b
J1 rep2
J1 rep1
TT2 rep2
TT2 rep1
GLN KO C43 P3
GLN KO C13 P3
GLN KO C43 P5
GLN KO C13 P5
c
GLN KO
C23 C25
TT2 (empty vector)
Ladder
KLHDC4
GAPDH
100kDa
70kDa
35kDa
d
KLHDC4 protein level (relative to GAPDH)
0.0052
0.0028
TT2 (empty vector)
C25
C23
GLN KO
e
5' ChIP primers
GLN
1,187bp
sgRNA-1
1,382bp
sgRNA-2
3,006bp
sgRNA-3
3,414bp
sgRNA-4
f
H3K27ac % of input
H3K9me3 % of input
Gapdh
IAP
dCas9 control
sgRNA-1
sgRNA-2
sgRNA-3
sgRNA-4
g
Promoter cloning site
pGL3-Enhancer Vector
Luciferase gene
polyA
SV40 enhancer
Enhancer cloning site
pGL3-Promoter Vector
SV40 minimal promoter
Luciferase gene
polyA

Figure S2: Deletion or silencing of polymorphic GLN leads to downregulation of *Klhdc4* through loss of *cis*-regulatory function

a) PCR validates the successful deletion of the polymorphic GLN element in TT2 cells. Two independent clones are included. Expected amplicons are indicated with arrows. *Gapdh* primer serves as positive control. The top schematic diagram indicates the position of designed sgRNAs and PCR primers. PCR experiments were repeated (n=3), with the figure showing a representative result. **b)** Smooth scatter plots show high correlation between RNA-seq from biological replicates. Genes with more than 10 reads in either of two replicates are plotted. The x and y axes are shown as log2(FPKM+1) in labelled cells. **c)** Western blot shows downregulation of KLHDC4 protein level in early passage GLN KO clones compared to empty vector control. GAPDH serves as loading control. Western blots were repeated with independently derived lysates (n=3). **d)** Quantitative comparison of western blot shows significant downregulation of KLHDC4 protein level in early passage GLN KO clones compared to empty vector control. The y axis indicates protein level of KLHDC4 relative to GAPDH and normalized to empty vector control. Error bars reflect standard deviations with the centers indicating the means of 3 technical replicates. Two-tailed T-test is used to calculate p-values. **e)** The schematic indicates the position of designed single guide RNAs (sgRNAs) used to target the dCas9-KRAB protein to the GLN element and the locations of the PCR primers. **f)** ChIP-qPCR of H3K27ac (top) and H3K9me3 (bottom) at *Gapdh* promoter and IAP control loci. *Gapdh* serves as positive control for H3K27ac and negative control for H3K9me3. Whereas IAP serves as negative control for H3K27ac and positive control for H3K9me3. Error bars reflect standard deviations with the centers indicating the means of 3 technical replicates. Note that IAP primers are designed based on the consensus sequence of IAP-D and target multiple proviruses within this subfamily. **g)** The schematic shows the structure of pGL3 enhancer and promoter plasmids.

Figure S3

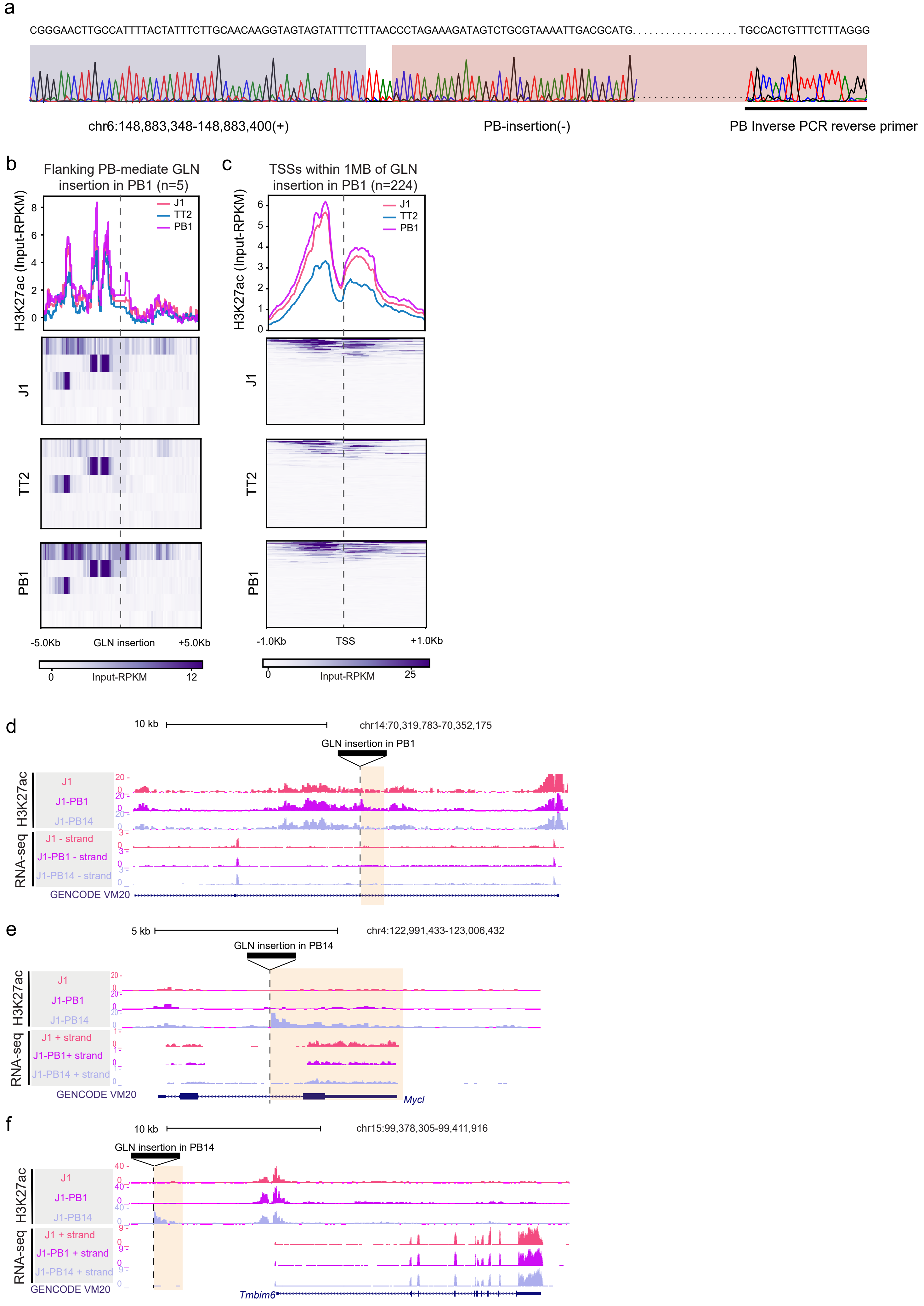

a
CGGGAACTTGCCATTTTACTATTTCTTGCAACAAGGTAGTAGTATTTCTTTAACCCTAGAAAGATAGTCTGCGTAAAATTGACGCATG. . . . . TGCCACTGTTTCTTTAGGG
chr6:148,883,348-148,883,400(+)
PB-insertion(-)
PB Inverse PCR reverse primer
b
Flanking PB-mediate GLN insertion in PB1 (n=5)
H3K27ac (Input-RPKM)
J1
TT2
PB1
-5.0Kb
GLN insertion
+5.0Kb
0
Input-RPKM
12
c
TSSs within 1MB of GLN insertion in PB1 (n=224)
H3K27ac (Input-RPKM)
J1
TT2
PB1
-1.0Kb
TSS
+1.0Kb
0
Input-RPKM
25
d
10 kb
chr14:70,319,783-70,352,175
GLN insertion in PB1
H3K27ac
RNA-seq
J1
J1-PB1
J1-PB14
J1 - strand
J1-PB1 - strand
J1-PB14 - strand
GENCODE VM20
e
5 kb
chr4:122,991,433-123,006,432
GLN insertion in PB14
H3K27ac
RNA-seq
J1
J1-PB1
J1-PB14
J1 + strand
J1-PB1+ strand
J1-PB14 + strand
GENCODE VM20
Mycl
f
10 kb
chr15:99,378,305-99,411,916
GLN insertion in PB14
H3K27ac
RNA-seq
J1
J1-PB1
J1-PB14
J1 + strand
J1-PB1 + strand
J1-PB14 + strand
GENCODE VM20
Tmbim6

Figure S3: GLN element can shape nearby epigenetic and transcriptional states in a position-dependent manner

a) Representative Sanger sequencing result of an inverse PCR amplicon defines a GLN insertion position in PB14. The DNA sequence from PB513Re vector and endogenous genome is labelled with red and blue, respectively. **b-c)** Aggregation plots and heatmaps show H3K27ac ChIP-seq (Input-RPKM) signal at regions ±5 kb of GLN insertions (n=5) (**b**) and transcription start sites within 1Mb of GLN insertions (n=244) (**c**) in PB1 compared to J1 and TT2. While a subset of new elements gain activity, others are not enriched with H3K27ac. **d-f)** Genome browser screenshots of validated PB-mediated GLN insertions shows the associated increase of H3K27ac signal at nearby regions (orange shading).

Figure S4

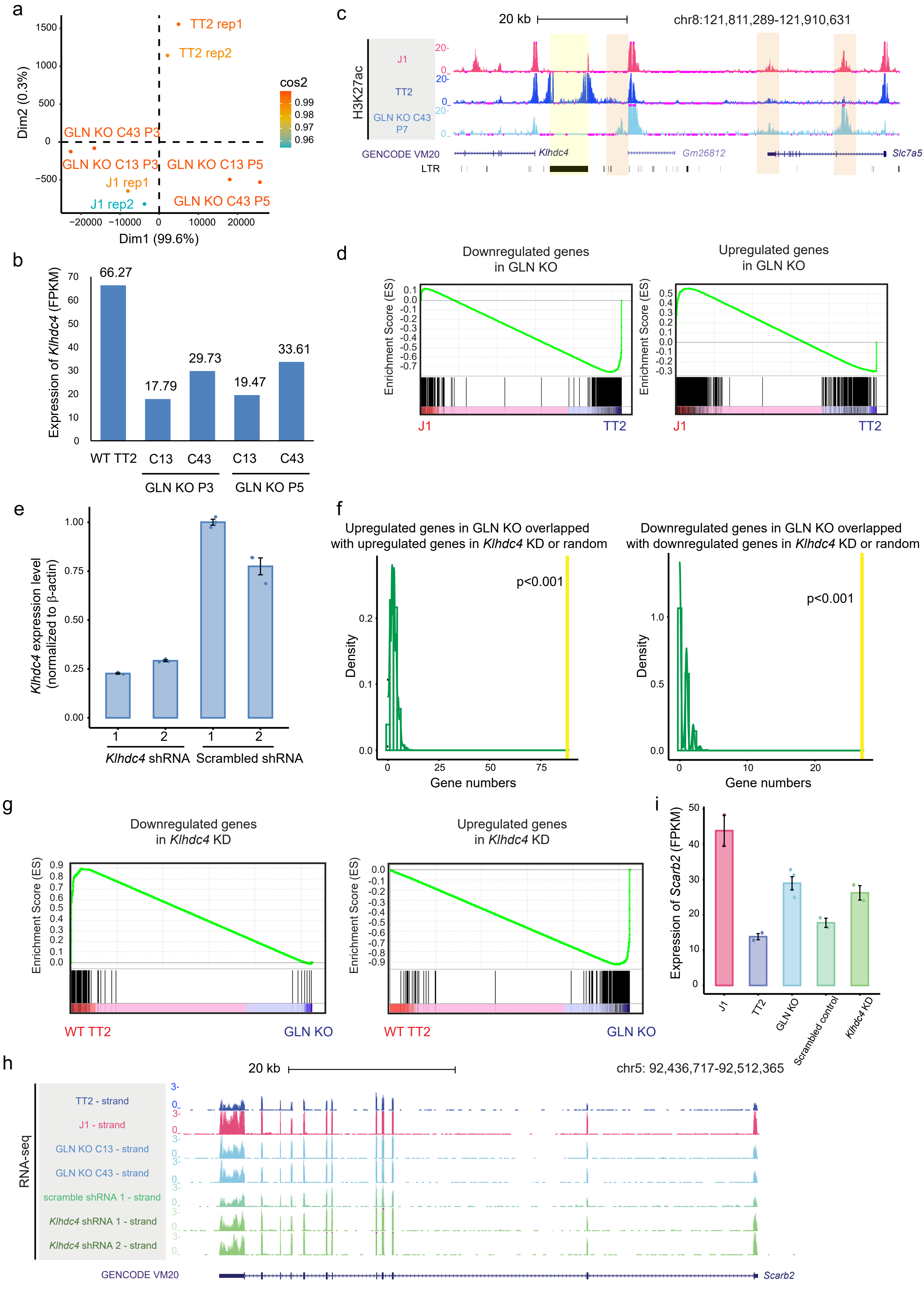

a
Dim2 (0.3%)
Dim1 (99.6%)
cos2
0.99
0.98
0.97
0.96
TT2 rep1
TT2 rep2
GLN KO C43 P3
GLN KO C13 P3
GLN KO C13 P5
J1 rep1
J1 rep2
GLN KO C43 P5
b
Expression of Klhdc4 (FPKM)
66.27
17.79
29.73
19.47
33.61
WT TT2
C13
C43
C13
C43
GLN KO P3
GLN KO P5
c
20 kb
chr8:121,811,289-121,910,631
H3K27ac
J1
TT2
GLN KO C43 P7
GENCODE VM20
Klhdc4
Gm26812
Slc7a5
LTR
d
Downregulated genes in GLN KO
Upregulated genes in GLN KO
Enrichment Score (ES)
J1
TT2
e
Klhdc4 expression level (normalized to β-actin)
Klhdc4 shRNA
Scrambled shRNA
f
Upregulated genes in GLN KO overlapped with upregulated genes in Klhdc4 KD or random
Downregulated genes in GLN KO overlapped with downregulated genes in Klhdc4 KD or random
p<0.001
p<0.001
Density
Gene numbers
g
Downregulated genes in Klhdc4 KD
Upregulated genes in Klhdc4 KD
Enrichment Score (ES)
WT TT2
GLN KO
i
Expression of Scarb2 (FPKM)
J1
TT2
GLN KO
Scrambled control
Klhdc4 KD
h
20 kb
chr5: 92,436,717-92,512,365
RNA-seq
TT2 - strand
J1 - strand
GLN KO C13 - strand
GLN KO C43 - strand
scramble shRNA 1 - strand
Klhdc4 shRNA 1 - strand
Klhdc4 shRNA 2 - strand
GENCODE VM20
Scarb2

Figure S4: The polymorphic GLN element contributes to strain-specific transcriptomic patterns

a) Principal components analysis (PCA) reveals closer relationship between transcriptomes of GLN KO clones and J1. The colors for individual lines are adjusted by the qualities of representation. **b)** Bar chart shows slightly increased expression (FPKM) of *Klhdc4* in late passage GLN KO clones compared to early passage GLN KO clones. **c)** A screenshot shows the dysregulated H3K27ac peaks (orange shading) upon GLN (yellow shading) KO. Notably, two peaks with increased signal locating in *Slc7a5* is correlated to the increased signal in WT J1 compared to WT TT2. **d)** Gene set enrichment analysis (GSEA) reveals that the dysregulated genes in GLN KO are significantly enriched for sets of differentially expressed genes in TT2 versus J1. Downregulated genes in GLN KO are correlated with higher expression in TT2 (left), whereas upregulated genes are correlated with higher expression in J1 (right). **e)** RT-qPCR validates efficiency of *Klhdc4* knockdown. Normalized expression, relative to *β-actin*, for cells transduced with *Klhdc4* shRNA is compared to scrambled control. Error bars reflect standard deviations with the centers indicating the means of 3 technical replicates. **f)** Histograms/density curves indicate the distribution of overlap between randomly selected genes (selected 1,000 times) and upregulated (n=647) (left) and downregulated (n=310) (right) genes upon GLN KO. The yellow lines show the actual number of upregulated (n=88) and downregulated (n=27) genes that are commonly defined with significantly upregulated (n=218) and downregulated (n=92) in *Klhdc4* KD cells, respectively. Significant enrichment of dysregulated genes upon GLN KO among genes with concordant change in *Klhdc4* KD cells. One-tailed p-values without multiple comparison adjustments are obtained via non-parametric bootstrapping. **g)** GSEA shows that the dysregulated genes in *Klhdc4* KD are significantly enriched for sets of differentially expressed genes in GLN KO cells. Downregulated genes upon *Klhdc4* KD are correlated with reduced expression in GLN KO (left), whereas upregulated genes are correlated with higher expression in GLN KO cells (right). **h)** A genome browser screenshot of stranded RNA-seq (RPM) illustrates upregulation of the *Scarb2* gene upon *Klhdc*4 KD or GLN KO, which shows similarly higher expression in J1 compared to TT2. **i)** Bar chart indicates higher RNA-seq signal (FPKM) of *Scarb2* gene in WT J1, GLN KO, and *Klhdc4* KD mESCs, compared to WT TT2 and scrambled control cells. Error bars reflect the standard deviations of biological replicates for GLN KO cells (n=4) and all other samples (n=2). The center of the error bars indicates the mean of technical replicates.

Figure S5

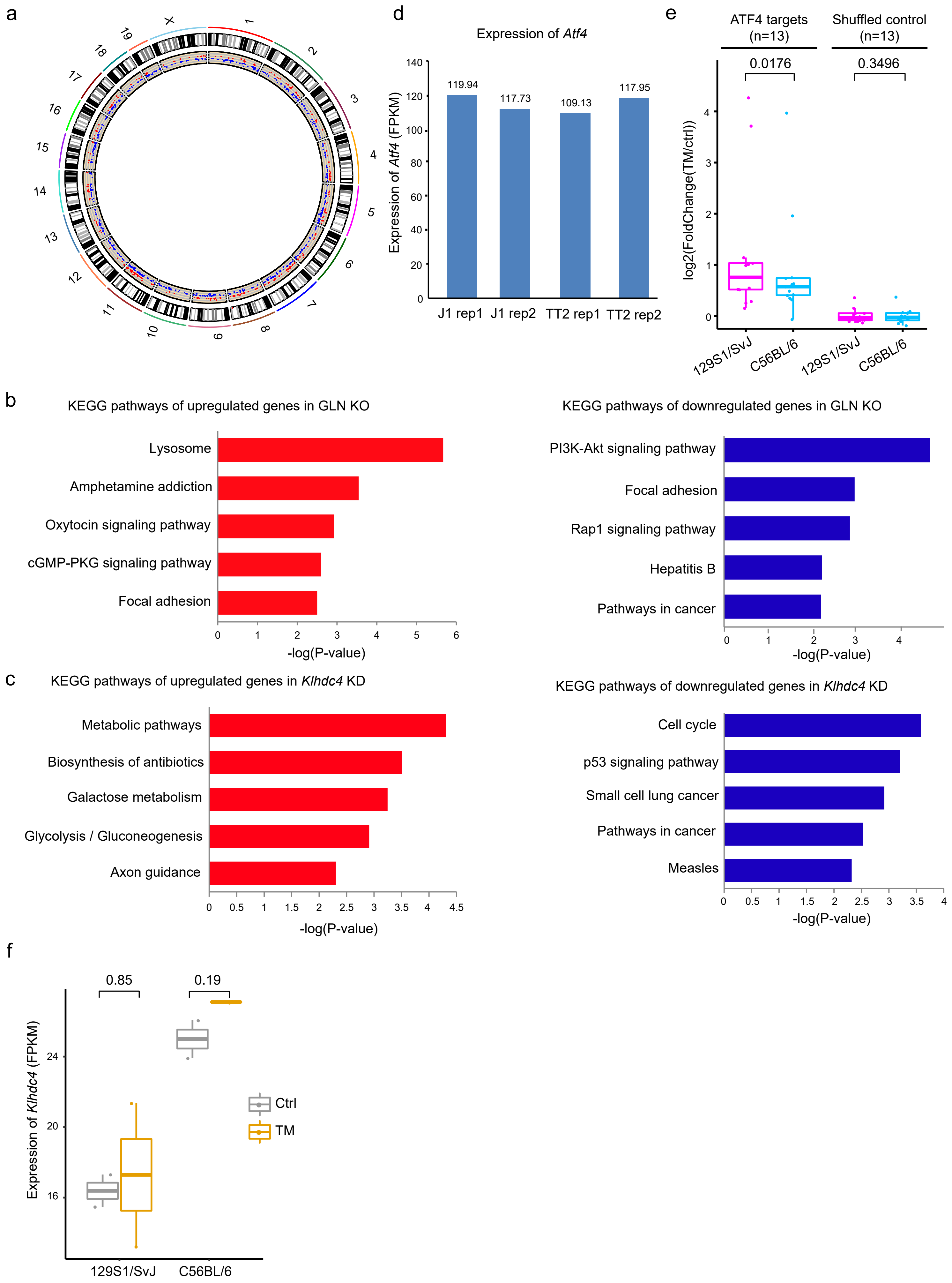
a
1
2
3
4
5
6
7
8
9
10
11
12
13
14
15
16
17
18
19
X
d
Expression of Atf4
Expression of Atf4 (FPKM)
140
120
100
80
60
40
20
0
119.94
117.73
109.13
117.95
J1 rep1
J1 rep2
TT2 rep1
TT2 rep2
e
ATF4 targets (n=13)
Shuffled control (n=13)
0.0176
0.3496
log2(FoldChange(TM/ctrl))
4
3
2
1
0
129S1/SvJ
C56BL/6
129S1/SvJ
C56BL/6
b
KEGG pathways of upregulated genes in GLN KO
Lysosome
Amphetamine addiction
Oxytocin signaling pathway
cGMP-PKG signaling pathway
Focal adhesion
0
1
2
3
4
5
6
-log(P-value)
KEGG pathways of downregulated genes in GLN KO
PI3K-Akt signaling pathway
Focal adhesion
Rap1 signaling pathway
Hepatitis B
Pathways in cancer
0
1
2
3
4
-log(P-value)
c
KEGG pathways of upregulated genes in Klhdc4 KD
Metabolic pathways
Biosynthesis of antibiotics
Galactose metabolism
Glycolysis / Gluconeogenesis
Axon guidance
0
0.5
1
1.5
2
2.5
3
3.5
4
4.5
-log(P-value)
KEGG pathways of downregulated genes in Klhdc4 KD
Cell cycle
p53 signaling pathway
Small cell lung cancer
Pathways in cancer
Measles
0
0.5
1
1.5
2
2.5
3
3.5
4
-log(P-value)
f
0.85
0.19
Expression of Klhdc4 (FPKM)
24
20
16
Ctrl
TM
129S1/SvJ
C56BL/6

Figure S5: Polymorphic GLN regulates transcriptional network through ATF4

a) Circos plot shows the genomic locations of dysregulated genes upon GLN deletion. Downregulated (n=310) and upregulated genes (n=647) upon GLN KO are labelled with red and blue, respectively. Results indicate that dysregulated genes are spread throughout the genome. No dysregulated genes are found within 1Mb of the polymorphic GLN element other than *Klhdc4*. **b-c)** The most significantly enriched KEGG pathways among upregulated (left) and downregulated (right) genes upon GLN KO **(b)** and *Klhdc4* KD **(c)** are shown. The negative log10-transformed one-tailed P values obtained from Fisher's Exact test for each pathway are plotted in the bar charts. **d)** Bar chart shows unaltered transcriptional levels (FPKM) of *Atf4* in WT TT2 and WT J1 cell lines. **e)** Boxplot demonstrates the higher fold change of ATF4-targets (n=13) expression upon TM treatment in MEFs derived from 129S1/SvJ than C56BL/6 strain. The center and bounds of boxes refer to the median and quartile of all the dots, respectively. The minima and maxima of boxplots indicate Quartile 1 - 1.5 x Interquartile range and Quartile 3 + 1.5 x Interquartile range, respectively. One-tailed paired T-test is used to measure significance. **f)** Boxplot shows tunicamycin (TM) treatment does not result in differential expression of *Klhdc4* in MEFs derived from 129S1/SvJ and C56BL/6 strains (n=2 for each condition). The center and bounds of boxes refer to the median and quartile of all the dots, respectively. The minima and maxima of boxplots indicate Quartile 1 - 1.5 x Interquartile range and Quartile 3 + 1.5 x Interquartile range, respectively. One-tailed T-test is used to measure the significance.

Figure S6

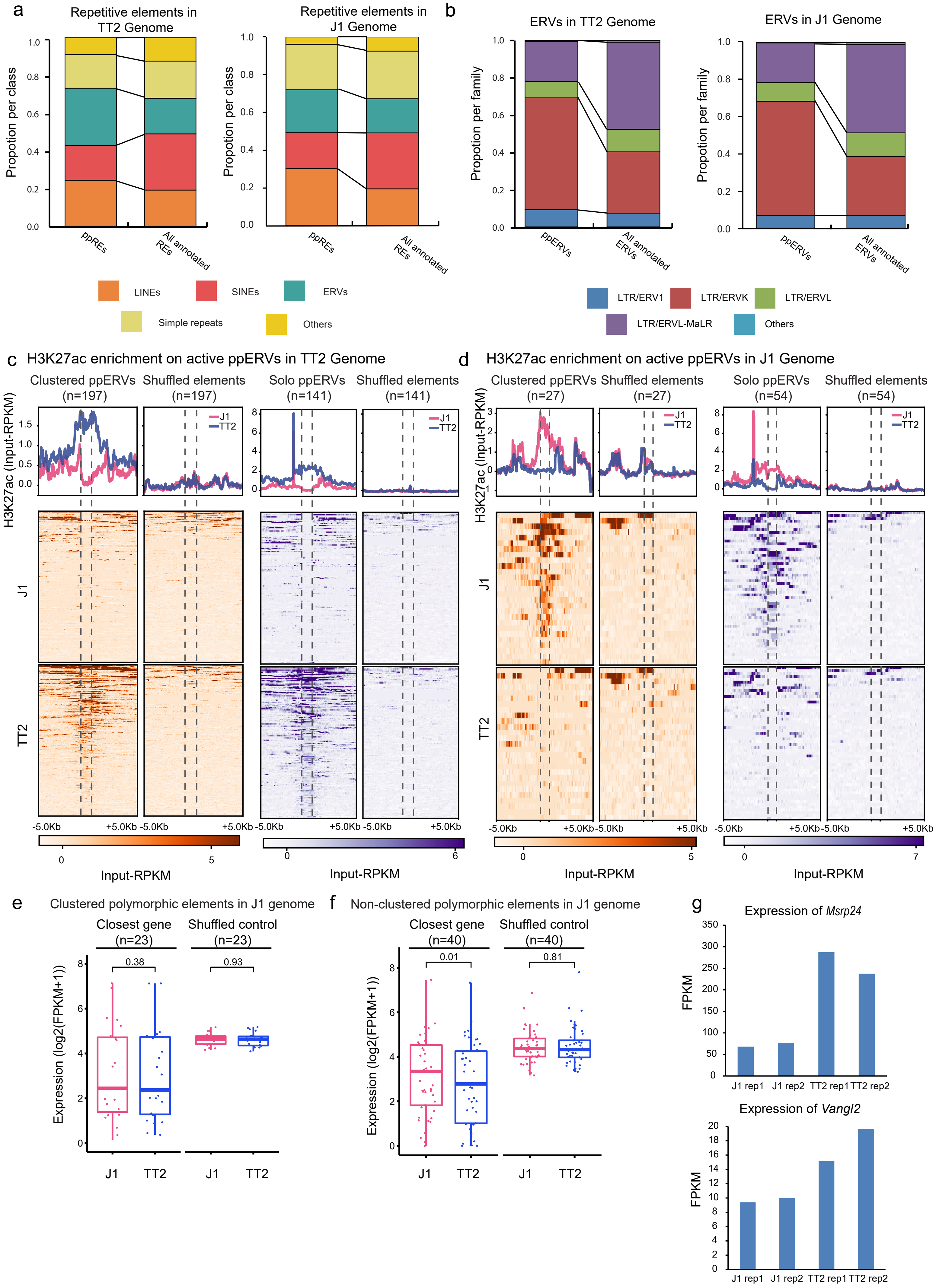
a
Repetitive elements in TT2 Genome
Repetitive elements in J1 Genome
Propotion per class
ppREs
All annotated REs
LINEs
SINEs
ERVs
Simple repeats
Others
b
ERVs in TT2 Genome
ERVs in J1 Genome
Propotion per family
ppERVs
All annotated ERVs
LTR/ERV1
LTR/ERVK
LTR/ERVL
LTR/ERVL-MaLR
Others
c H3K27ac enrichment on active ppERVs in TT2 Genome
Clustered ppERVs (n=197)
Shuffled elements (n=197)
Solo ppERVs (n=141)
Shuffled elements (n=141)
d H3K27ac enrichment on active ppERVs in J1 Genome
Clustered ppERVs (n=27)
Shuffled elements (n=27)
Solo ppERVs (n=54)
Shuffled elements (n=54)
H3K27ac (Input-RPKM)
J1
TT2
-5.0Kb
+5.0Kb
Input-RPKM
e
Clustered polymorphic elements in J1 genome
Closest gene (n=23)
Shuffled control (n=23)
0.38
0.93
Expression (log2(FPKM+1))
f
Non-clustered polymorphic elements in J1 genome
Closest gene (n=40)
Shuffled control (n=40)
0.01
0.81
g
Expression of Msrp24
Expression of Vangl2
FPKM
J1 rep1
J1 rep2
TT2 rep1
TT2 rep2

Figure S6: Genome-wide analysis identifies additional polymorphic ERVs capable of epigenomic and transcriptomic regulation

a) Stacked bar charts show the distribution of repetitive element (RE) classes among ppREs in TT2 (left) and J1 (right) genome as well as all annotated REs in the corresponding reference assemblies. A moderate but significant overrepresentation (TT2: $p<1 \times 10^{-22}$; J1: $p<1 \times 10^{-22}$, from two-tailed hypergeometric distribution) of ERVs is detected in both lines. **b)** Stacked bar charts show the distribution of ERV families among ppERVs and all annotated ERVs in TT2 (left) and J1 (right) genomes in the corresponding reference assemblies. ERV-K is significantly overrepresented (TT2: $p<1 \times 10^{-22}$; J1: $p<1 \times 10^{-22}$, from two-tailed hypergeometric distribution) in both TT2 and J1 genomes. **c-d)** Aggregation plots and heatmaps show both active clustered polymorphic elements (left) and solo polymorphic elements (right) in TT2 **(c)** and J1 **(d)** mESCs are associated with higher H3K27ac enrichment within ±5 kb regions in corresponding cell lines. **e)** Boxplots show no significant difference in the expression of the closest genes to clustered polymorphic elements in J1 cells. The center and bounds of boxes refer to the median and quartile of all the dots, respectively. The minima and maxima of boxplots indicate Quartile 1 - 1.5 x Interquartile range and Quartile 3 + 1.5 x Interquartile range, respectively. This may result from the small number of polymorphic clusters defined in the J1 genome. One-tailed paired T-test was applied to calculate P-values. **f)** Boxplots indicate solo polymorphic elements in J1 are associated with significantly higher expression of their closest genes in J1 compared to TT2 cells. The center and bounds of boxes refer to the median and quartile of all the dots, respectively. The minima and maxima of boxplots indicate Quartile 1 - 1.5 x Interquartile range and Quartile 3 + 1.5 x Interquartile range, respectively. One-tailed paired T-test was applied to calculate P-values. **g)** Bar charts indicate higher RNA-seq signal (FPKM) of *Msrp24* and *Vangl2* genes in TT2 than J1 mESCs.

Table S1: Genomic coordinates of validated PiggyBac-mediated GLN insertions PB1

chromosome	coordinate	strand
chr13	13955360	-
chr14	70339071	-
chr14	73350178	-
chr16	89264643	-
chr3	86923864	+
**PB14**		
**chromosome**	**coordinate**	**strand**
chr1	161151575	+
chr10	98711683	+
chr13	29791768	+
chr13	13955360	-
chr14	80867605	+
chr14	59203500	-
chr14	80868204	+
chr15	99385305	-
chr3	86923909	-
chr3	32764219	+
chr3	152738359	+
chr4	122998933	-
chr6	119460481	+
chr6	148883396	-
chr6	90950194	-
chr9	114345333	-

Table S2: All antibodies used in this study

**Antibody**	**Assay**	**Vendor (Cat #)**	**Dilution factor or amount used**
Histone H3K27ac antibody (pAb)	ChIP	Active motif (39133)	5μg/IP
Anti-Histone H3K9me3 antibody	ChIP	Abcam(ab8898)	5μg/IP
KLHDC4 Polyclonal Antibody	Western blot	Life Technologies (PA5-59669)	1: 1000
Anti-GAPDH antibody	Western blot	Abcam(ab8245)	1: 10,000
Goat Anti-Rabbit IgG H&L (HRP)	Western blot	Abcam(ab205718)	1:20,000
Goat Anti-Mouse IgG H&L (HRP) preadsorbed	Western blot	Abcam(ab97040)	1:10,000

Table S3: Vectors used in this study

Vector	Assay	Digestion enzyme	Incubation temperature
PX330A-1x2-GFP	CRISPR-Cas9 deletion	BbsI; BsaI (NEB)	37˚C; 37˚C
PX330S-2	CRISPR-Cas9 deletion	BbsI; BsaI (NEB)	37˚C; 37˚C
pSIH1-H1-copGFP shRNA Expression Lentivector	shRNA knockdown	BamHI/EcoRI-HF (NEB)	37˚C
pL-CRISPR.EFS.GFP	CRISPRi	BsmBI (NEB)	37˚C
PB513Re	PiggyBac	BamHI/EcoRI-HF (NEB)	37˚C
pGL3 enhancer vector	luciferase assay	BglII (NEB)	37˚C
pGL3 promoter vector	luciferase assay	BamHI (NEB)	37˚C

Table S4: Sequencing information for NGS datasets

	Sequencing platform	Library type	Read length	Sequencing depth
ChIP-seq	NextSeq 500	Paired end	75bp	~6-7 X
RNA-seq	NextSeq 500	Paired end	75bp	~70-100 M reads
WGS	MGISEQ-2000-RS	Paired end	200bp	~130 X

Supplemental References:

1. Tamura, K. *et al.* Estimating divergence times in large molecular phylogenies. *Proc Natl Acad Sci U S A* **109**, 19333-8 (2012).
2. Tamura, K., Tao, Q. & Kumar, S. Theoretical Foundation of the RelTime Method for Estimating Divergence Times from Variable Evolutionary Rates. *Mol Biol Evol* **35**, 1770-1782 (2018).
3. Nei, M. & Kumar, S. *Molecular evolution and phylogenetics*, (Oxford university press, 2000).
4. Kumar, S., Stecher, G., Li, M., Knyaz, C. & Tamura, K. MEGA X: Molecular Evolutionary Genetics Analysis across Computing Platforms. *Mol Biol Evol* **35**, 1547-1549 (2018).
