# Supplementary material for: Mouse strain-specific polymorphic provirus functions as cis-regulatory element leading to epigenomic and transcriptomic variations
Source: Nat Commun. 2021 Nov 9;12:6462. doi: 10.1038/s41467-021-26630-z (PMC8578388; doi:10.1038/s41467-021-26630-z)
Supplement: Supplementary file 2 — Description of additional Supplementary File [file 41467_2021_26630_MOESM2_ESM.pdf]

Description for Additional Supplementary Files

File Name: Supplementary Data Table 1
Description: TF binding motifs found on Polymorphic GLN LTR sequence

File Name: Supplementary Data Table 2
Description: Commonly downregulated genes in GLN KO and Klhdc4 KD cells

File Name: Supplementary Data Table 3
Description: List of ppERVs by subfamily

File Name: Supplementary Data Table 4
Description: List of all primers used in this study
