# Supplementary material for: Mouse strain-specific polymorphic provirus functions as cis-regulatory element leading to epigenomic and transcriptomic variations
Source: Nat Commun. 2021 Nov 9;12:6462. doi: 10.1038/s41467-021-26630-z (PMC8578388; doi:10.1038/s41467-021-26630-z)
Supplement: Supplementary file 7 — Reporting Summary [file 41467_2021_26630_MOESM7_ESM.pdf]

nature research

Corresponding author(s): Danny Chi Yeu Leung

Last updated by author(s): 26/09/21

Reporting Summary

Nature Research wishes to improve the reproducibility of the work that we publish. This form provides structure for consistency and transparency in reporting. For further information on Nature Research policies, see our Editorial Policies and the Editorial Policy Checklist.

Statistics

For all statistical analyses, confirm that the following items are present in the figure legend, table legend, main text, or Methods section.

n/a	Confirmed	
☐	☒	The exact sample size (*n*) for each experimental group/condition, given as a discrete number and unit of measurement
☐	☒	A statement on whether measurements were taken from distinct samples or whether the same sample was measured repeatedly
☐	☒	The statistical test(s) used AND whether they are one- or two-sided<br>*Only common tests should be described solely by name; describe more complex techniques in the Methods section.*
☒	☐	A description of all covariates tested
☐	☒	A description of any assumptions or corrections, such as tests of normality and adjustment for multiple comparisons
☐	☒	A full description of the statistical parameters including central tendency (e.g. means) or other basic estimates (e.g. regression coefficient) AND variation (e.g. standard deviation) or associated estimates of uncertainty (e.g. confidence intervals)
☐	☒	For null hypothesis testing, the test statistic (e.g. *F*, *t*, *r*) with confidence intervals, effect sizes, degrees of freedom and *P* value noted<br>*Give P values as exact values whenever suitable.*
☒	☐	For Bayesian analysis, information on the choice of priors and Markov chain Monte Carlo settings
☒	☐	For hierarchical and complex designs, identification of the appropriate level for tests and full reporting of outcomes
☐	☒	Estimates of effect sizes (e.g. Cohen's *d*, Pearson's *r*), indicating how they were calculated

Our web collection on statistics for biologists contains articles on many of the points above.

Software and code

Policy information about availability of computer code

Data collection: No software was used for data collection

Data analysis: The software and versions used in this study are as follows:

MEGA-X 10.1.8
Bowtie 2.3.5.1
Picard 2.3.0
SAMTools 1.3.1
BEDTools v2.27.1
STAR _2.6.1a
RSEM v1.3.0
DESeq2 _1.20.0
GSEA 3.0
HOMER-4.9.1-6
DAVID v6.8
TFBIND online tool （with ID from TRANSFAC R.3.4） (https:// https://tfbind.hgc.jp/)
MACS2 2.1.1.20160309
deeptools 3.0.2
QUAST v5.0.2
sva 3.40.0
MUMmer v4.0.0rc1

For manuscripts utilizing custom algorithms or software that are central to the research but not yet described in published literature, software must be made available to editors and reviewers. We strongly encourage code deposition in a community repository (e.g. GitHub). See the Nature Research guidelines for submitting code & software for further information.

Data

Policy information about availability of data

All manuscripts must include a data availability statement. This statement should provide the following information, where applicable:

- Accession codes, unique identifiers, or web links for publicly available datasets
- A list of figures that have associated raw data
- A description of any restrictions on data availability

All the sequencing data generated in this study have been deposited in the National Center for Biotechnology Information (NCBI) database under GEO accession code GSE165214 [https://www.ncbi.nlm.nih.gov/geo/query/acc.cgi?acc=GSE165214]. The consensus sequence of GLN used in this study was obtained from Dfam database [https://dfam.org/home]. Source data are provided with this paper. All previously published datasets used in our integrative analyses are found in the NCBI GEO database under the following accession codes: RNA-seq datasets of murine liver are (GSE45684) [https://www.ncbi.nlm.nih.gov/geo/query/acc.cgi?acc=GSE45684], RNA-seq datasets from C57BL/6 mouse tissues (GSE29184) [https://www.ncbi.nlm.nih.gov/geo/query/acc.cgi?acc=GSE29184], RNA-seq datasets of 129S1/SvJ and C57BL/6 mice treated with DMSO and/or tunicamycin (GSE63756) [https://www.ncbi.nlm.nih.gov/geo/query/acc.cgi?acc=GSE63756], and H3K9me3 ChIP-seq datasets

Field-specific reporting

Please select the one below that is the best fit for your research. If you are not sure, read the appropriate sections before making your selection.

[x] Life sciences [ ] Behavioural & social sciences [ ] Ecological, evolutionary & environmental sciences

For a reference copy of the document with all sections, see nature.com/documents/nr-reporting-summary-flat.pdf

Life sciences study design

All studies must disclose on these points even when the disclosure is negative.

Sample size	For all epigenomic and transcriptomic assays, biological replicates (n≥2) were analyzed to ensure reproducibility. Further validation experiments were conducted on independent biological replicates. For all other experiments biological replicates (n≥2) and technical replicates (n≥3) were conducted each time to account for variance. Experiments were also repeated at least twice to ensure reproducibility.
Data exclusions	No data was excluded from analyses
Replication	For all epigenomic and transcriptomic experiments, technical replicates (n≥2) were conducted. All replicates were compared to ensure concordance of datasets. For knockout, knockdown, CRISPRi, and PiggyBac transposon experiments at least 2 independent clones were analyzed. All results were included in the manuscript.
Randomization	Randomization was not necessary for epigenomics studies of this type. As the samples are divided by their genetic backgrounds, they cannot be randomized.
Blinding	Investigators were not blinded to the group allocations as they samples are divided by their genetic backgrounds.

Reporting for specific materials, systems and methods

We require information from authors about some types of materials, experimental systems and methods used in many studies. Here, indicate whether each material, system or method listed is relevant to your study. If you are not sure if a list item applies to your research, read the appropriate section before selecting a response.

Materials & experimental systems

n/a	Involved in the study
☐	☒ Antibodies
☐	☒ Eukaryotic cell lines
☒	☐ Palaeontology and archaeology
☒	☐ Animals and other organisms
☒	☐ Human research participants
☒	☐ Clinical data
☒	☐ Dual use research of concern

Methods

n/a	Involved in the study
☐	☒ ChIP-seq
☒	☐ Flow cytometry
☒	☐ MRI-based neuroimaging

Antibodies

Antibodies used

ChIP-seq: H3K27ac (Active motif(39133)); H3K9me3 (Abcam(ab8898)).
Western blot: Primary antibodies: KLHDC4 (Thermo Fisher (PA5-59669)); GAPDH (Abcam(ab8245)). Secondary antibodies: Goat Anti-Rabbit IgG H&L (HRP)(Abcam(ab97040)); Goat Anti-Mouse IgG H&L (HRP) preadsorbed (Abcam(ab97040))

Validation

Peptide arrays and western blots have been carried out to validate specificity of histone modification antibodies. Product information can be found https://www.activemotif.com/catalog/details/39133 and https://www.abcam.com/histone-h3-tri-methyl-k9-antibody-chip-grade-ab8898.html.
For KLHDC4 and GAPDH antibodies, product information can be found at https://www.thermofisher.com/antibody/product/KLHDC4-Antibody-Polyclonal/PA5-59669 and https://www.abcam.com/gapdh-antibody-6c5-loading-control-ab8245.html. No additional validations experiments were conducted for the these antibodies.

Eukaryotic cell lines

Policy information about cell lines

Cell line source(s)

TT2 and J1 mESC lines. Lines were received from Dr Yoichi Shinkai from the RIKEN
HEK293 cells were used for packaging lentiviruses. Cells were received for Dr Bing Ren from UCSD

Authentication

No additional authentication was done

Mycoplasma contamination

Cell lines tested negative for mycoplasma

Commonly misidentified lines
(See ICLAC register)

No commonly misidentified cell lines were used in the study

ChIP-seq

Data deposition

☒ Confirm that both raw and final processed data have been deposited in a public database such as GEO.

☒ Confirm that you have deposited or provided access to graph files (e.g. BED files) for the called peaks.

Data access links
May remain private before publication.

https://www.ncbi.nlm.nih.gov/geo/query/acc.cgi?acc=GSE165214

Files in database submission

Raw files:
ChIPseq_H3K27ac_J1.fastq.gz
ChIPseq_H3K27ac_TT2.fastq.gz
ChIPseq_H3K27ac_J1_GLN_PB1.fastq.gz
ChIPseq_H3K27ac_J1_GLN_PB14.fastq.gz
ChIPseq_Input_J1.fastq.gz
ChIPseq_Input_TT2.fastq.gz
ChIPseq_Input_J1_PB1.fastq.gz
ChIPseq_Input_J1_PB14.fastq.gz
RNAseq_J1_rep1.R1.fastq.gz
RNAseq_J1_rep2.R1.fastq.gz
RNAseq_TT2_rep1.R1.fastq.gz
RNAseq_TT2_rep2.R1.fastq.gz
RNAseq_TT2_GLN_KO_C13_P3.R1.fastq.gz
RNAseq_TT2_GLN_KO_C43_P3.R1.fastq.gz

RNAseq_TT2_GLN_KO_C13_P5.R1.fastq.gz
RNAseq_TT2_GLN_KO_C43_P5.R1.fastq.gz
RNAseq_TT2_GLN_KD_shRNA_1.R1.fastq.gz
RNAseq_TT2_GLN_KD_shRNA_2.R1.fastq.gz
RNAseq_TT2_GLN_KD_scramble_shRNA_1.R1.fastq.gz
RNAseq_TT2_GLN_KD_scramble_shRNA_2.R1.fastq.gz
RNAseq_TT2_Klhdc4_KD_shRNA_1.R1.fastq.gz
RNAseq_TT2_Klhdc4_KD_shRNA_2.R1.fastq.gz
RNAseq_TT2_Klhdc4_KD_scramble_shRNA_1.R1.fastq.gz
RNAseq_J1_GLN_PB1.R1.fastq.gz
RNAseq_J1_GLN_PB14.R1.fastq.gz
RNAseq_J1_rep1.R2.fastq.gz
RNAseq_J1_rep2.R2.fastq.gz
RNAseq_TT2_rep1.R2.fastq.gz
RNAseq_TT2_rep2.R2.fastq.gz
RNAseq_TT2_GLN_KO_C13_P3.R2.fastq.gz
RNAseq_TT2_GLN_KO_C43_P3.R2.fastq.gz
RNAseq_TT2_GLN_KO_C13_P5.R2.fastq.gz
RNAseq_TT2_GLN_KO_C43_P5.R2.fastq.gz
RNAseq_TT2_GLN_KD_shRNA_1.R2.fastq.gz
RNAseq_TT2_GLN_KD_shRNA_2.R2.fastq.gz
RNAseq_TT2_GLN_KD_scramble_shRNA_1.R2.fastq.gz
RNAseq_TT2_GLN_KD_scramble_shRNA_2.R2.fastq.gz
RNAseq_TT2_Klhdc4_KD_shRNA_1.R2.fastq.gz
RNAseq_TT2_Klhdc4_KD_shRNA_2.R2.fastq.gz
RNAseq_TT2_Klhdc4_KD_scramble_shRNA_1.R2.fastq.gz
RNAseq_J1_GLN_PB1.R2.fastq.gz
RNAseq_J1_GLN_PB14.R2.fastq.gz
WGS_J1_R1.fastq.gz
WGS_TT2_R1.fastq.gz
WGS_J1_R2.fastq.gz
WGS_TT2_R2.fastq.gz
RNAseq_TT2_Klhdc4_KD_scramble_shRNA_2.R1.fastq.gz
RNAseq_TT2_Klhdc4_KD_scramble_shRNA_2.R2.fastq.gz
ChIPseq_H3K27ac_J1_paired_end.R1.fastq.gz
ChIPseq_H3K27ac_J1_paired_end.R2.fastq.gz
ChIPseq_H3K27ac_TT2_paired_end.R1.fastq.gz
ChIPseq_H3K27ac_TT2_paired_end.R2.fastq.gz
ChIPseq_H3K27ac_J1_GLN_PB1_paired_end.R1.fastq.gz
ChIPseq_H3K27ac_J1_GLN_PB1_paired_end.R2.fastq.gz
ChIPseq_H3K27ac_J1_GLN_PB14_paired_end.R1.fastq.gz
ChIPseq_H3K27ac_J1_GLN_PB14_paired_end.R2.fastq.gz
ChIPseq_H3K27ac_TT2_GLN_KO_C43_P7_paied_end.R1.fastq.gz
ChIPseq_H3K27ac_TT2_GLN_KO_C43_P7_paied_end.R2.fastq.gz
ChIPseq_input_J1_paired_end.R1.fastq.gz
ChIPseq_input_J1_paired_end.R2.fastq.gz
ChIPseq_input_TT2_paired_end.R1.fastq.gz
ChIPseq_input_TT2_paired_end.R2.fastq.gz
ChIPseq_input_J1_GLN_PB1_paired_end.R1.fastq.gz
ChIPseq_input_J1_GLN_PB1_paired_end.R2.fastq.gz
ChIPseq_input_J1_GLN_PB14_paired_end.R1.fastq.gz
ChIPseq_input_J1_GLN_PB14_paired_end.R2.fastq.gz
ChIPseq_input_TT2_GLN_KO_C43_P7_paied_end.R1.fastq.gz
ChIPseq_input_TT2_GLN_KO_C43_P7_paied_end.R2.fastq.gz

Processed files
H3K27ac_J1_Input_sub.rpkm.bw
H3K27ac_TT2_Input_sub.rpkm.bw
H3K27ac_J1_GLN_PB1_Input_sub.rpkm.bw
H3K27ac_J1_GLN_PB14_Input_sub.rpkm.bw
RNAseq_J1_rep1.str1.bw
RNAseq_J1_rep2.str1.bw
RNAseq_TT2_rep1.str1.bw
RNAseq_TT2_rep2.str1.bw
RNAseq_TT2_GLN_KO_C13_P3.str1.bw
RNAseq_TT2_GLN_KO_C43_P3.str1.bw
RNAseq_TT2_GLN_KO_C13_P5.str1.bw

RNAseq_TT2_GLN_KO_C43_P5.str1.bw
RNAseq_TT2_GLN_KD_shRNA_1.str1.bw
RNAseq_TT2_GLN_KD_shRNA_2.str1.bw
RNAseq_TT2_GLN_KD_scramble_shRNA_1.str1.bw
RNAseq_TT2_GLN_KD_scramble_shRNA_2.str1.bw
RNAseq_TT2_Klhdc4_KD_shRNA_1.str1.bw
RNAseq_TT2_Klhdc4_KD_shRNA_2.str1.bw
RNAseq_TT2_Klhdc4_KD_scramble_shRNA_1.str1.bw
RNAseq_J1_GLN_PB1.str1.bw
RNAseq_J1_GLN_PB14.str1.bw
RNAseq_J1_rep1.str2.bw
RNAseq_J1_rep2.str2.bw
RNAseq_TT2_rep1.str2.bw
RNAseq_TT2_rep2.str2.bw
RNAseq_TT2_GLN_KO_C13_P3.str2.bw
RNAseq_TT2_GLN_KO_C43_P3.str2.bw
RNAseq_TT2_GLN_KO_C13_P5.str2.bw
RNAseq_TT2_GLN_KO_C43_P5.str2.bw
RNAseq_TT2_GLN_KD_shRNA_1.str2.bw
RNAseq_TT2_GLN_KD_shRNA_2.str2.bw
RNAseq_TT2_GLN_KD_scramble_shRNA_1.str2.bw
RNAseq_TT2_GLN_KD_scramble_shRNA_2.str2.bw
RNAseq_TT2_Klhdc4_KD_shRNA_1.str2.bw
RNAseq_TT2_Klhdc4_KD_shRNA_2.str2.bw
RNAseq_TT2_Klhdc4_KD_scramble_shRNA_1.str2.bw
RNAseq_J1_GLN_PB1.str2.bw
RNAseq_J1_GLN_PB14.str2.bw
WGS_J1.bw.gz
WGS_TT2.bw.gz
RNAseq_TT2_Klhdc4_KD_scramble_shRNA_2.str1.bw
RNAseq_TT2_Klhdc4_KD_scramble_shRNA_2.str2.bw
H3K27ac_J1_paired_end_Input_sub.rpkm.bw
H3K27ac_TT2_paired_end_Input_sub.rpkm.bw
H3K27ac_J1_GLN_PB1_paired_end_Input_sub.rpkm.bw
H3K27ac_J1_GLN_PB14_paired_end_Input_sub.rpkm.bw
H3K27ac_TT2_GLN_KO_C43_P7_paied_end_Input_sub.rpkm.bw

Genome browser session
(e.g. UCSC)

https://genome.ucsc.edu/cgi-bin/hgTracks?db=mm10&lastVirtModeType=default&lastVirtModeExtraState=&virtModeType=default&virtMode=0&nonVirtPosition=&position=chr8%3A121763056%2D121862826&hgsid=1164745883_bta0nvV8M5KAaStyoDZeMiHzloB4

Methodology

Replicates	All epigenomics experiments were performed on at least 2 biological replicates.
Sequencing depth	ChIP-seq libraries were sequenced on the Illumina NextSeq platform with paired-end 75bp reads. All libraries were sequenced to greater than 20 million uniquely mapped reads.
Antibodies	ChIP-seq: H3K27ac (Active motif(39133)); H3K9me3 (Abcam(ab8898)).
Peak calling parameters	macs2 callpeak -f BED -g mm -q 0.05 --keep-dup all --nomodel
Data quality	Peak calls were used for confirmation of data quality. Comparison with previously published datasets from the same cell lines were compared.
Software	MACS2 2.1.1.20160309
