# Supplementary material for: Mouse strain-specific polymorphic provirus functions as cis-regulatory element leading to epigenomic and transcriptomic variations
Source: Nat Commun. 2021 Nov 9;12:6462. doi: 10.1038/s41467-021-26630-z (PMC8578388; doi:10.1038/s41467-021-26630-z)
Supplement: Supplementary file 8 — Source data [file 41467_2021_26630_MOESM8_ESM.zip › Source Data/Source Data_Figure 5b-Homer_result_upregulatedpromoters_upon GLN KO.html]

./down201\_homerout - Homer Known Motif Enrichment Results


# Homer Known Motif Enrichment Results (./down201\_homerout)

Homer *de novo* Motif Results  
Gene Ontology Enrichment Results  
Known Motif Enrichment Results (txt file)  
Total Target Sequences = 647, Total Background Sequences = 40267

|  |  |  |  |  |  |  |  |  |  |  |  |
| --- | --- | --- | --- | --- | --- | --- | --- | --- | --- | --- | --- |
| Rank | Motif | Name | P-value | log P-pvalue | q-value (Benjamini) | # Target Sequences with Motif | % of Targets Sequences with Motif | # Background Sequences with Motif | % of Background Sequences with Motif | Motif File | SVG |
| 1 | T A C G C T A G A T G C G A T C G T A C A G T C C T A G A G T C A G T C A G T C G T A C A G T C | Sp1(Zf)/Promoter/Homer | 1e-15 | -3.487e+01 | 0.0000 | 372.0 | 57.50% | 16831.4 | 41.80% | motif file (matrix) | svg |
| 2 | C T G A T C A G C A G T C T A G A C T G C T A G G A T C A T C G A C T G C T G A T C A G G A T C | Sp5(Zf)/mES-Sp5.Flag-ChIP-Seq(GSE72989)/Homer | 1e-10 | -2.355e+01 | 0.0000 | 583.0 | 90.11% | 32524.1 | 80.78% | motif file (matrix) | svg |
| 3 | G A C T T C A G C T A G A G T C A G T C G T A C A G T C C T G A A G T C A G T C A G T C G A C T A G T C A C T G A T G C | KLF3(Zf)/MEF-Klf3-ChIP-Seq(GSE44748)/Homer | 1e-9 | -2.246e+01 | 0.0000 | 446.0 | 68.93% | 22885.7 | 56.84% | motif file (matrix) | svg |
| 4 | C T A G A C T G C T A G T C A G T C A G T A C G C T A G A C T G | Maz(Zf)/HepG2-Maz-ChIP-Seq(GSE31477)/Homer | 1e-7 | -1.819e+01 | 0.0000 | 609.0 | 94.13% | 35203.4 | 87.43% | motif file (matrix) | svg |
| 5 | T G C A A G C T C A T G C G T A A G C T A C T G G A T C G T C A C G T A A G C T | Atf4(bZIP)/MEF-Atf4-ChIP-Seq(GSE35681)/Homer | 1e-6 | -1.513e+01 | 0.0000 | 129.0 | 19.94% | 5171.6 | 12.84% | motif file (matrix) | svg |
| 6 | C G T A C T A G A C T G A C T G G A C T C T A G C A G T C T A G C A T G G A T C | KLF5(Zf)/LoVo-KLF5-ChIP-Seq(GSE49402)/Homer | 1e-5 | -1.352e+01 | 0.0001 | 599.0 | 92.58% | 34894.1 | 86.66% | motif file (matrix) | svg |
| 7 | T C G A G C A T A C G T C T A G G T A C T C G A G C A T T G A C T C G A A C G T | Chop(bZIP)/MEF-Chop-ChIP-Seq(GSE35681)/Homer | 1e-5 | -1.208e+01 | 0.0003 | 97.0 | 14.99% | 3825.8 | 9.50% | motif file (matrix) | svg |
| 8 | G T A C C A G T A C T G A C T G A C T G G A T C A C T G A C G T A C T G A C T G A G T C G A T C | KLF6(Zf)/PDAC-KLF6-ChIP-Seq(GSE64557)/Homer | 1e-5 | -1.178e+01 | 0.0003 | 564.0 | 87.17% | 32488.7 | 80.69% | motif file (matrix) | svg |
| 9 | C T A G T C G A C G A T C T A G G C A T C A G T C T A G G A T C C G T A G T C A | CEBP:AP1(bZIP)/ThioMac-CEBPb-ChIP-Seq(GSE21512)/Homer | 1e-4 | -1.130e+01 | 0.0005 | 286.0 | 44.20% | 14521.4 | 36.07% | motif file (matrix) | svg |
| 10 | C T A G T C A G C A G T T C A G A C T G A C T G G A T C C T A G A C T G C T A G T C A G A T G C | KLF14(Zf)/HEK293-KLF14.GFP-ChIP-Seq(GSE58341)/Homer | 1e-4 | -9.507e+00 | 0.0027 | 625.0 | 96.60% | 37467.4 | 93.05% | motif file (matrix) | svg |
| 11 | T C A G T G A C G T A C T G C A G T A C C T A G G T A C A T G C A G T C G T C A A G T C G A C T | Klf9(Zf)/GBM-Klf9-ChIP-Seq(GSE62211)/Homer | 1e-2 | -6.268e+00 | 0.0627 | 362.0 | 55.95% | 20208.7 | 50.19% | motif file (matrix) | svg |
| 12 | T A G C T C A G C A T G G C A T A G C T C G A T A T G C C G T A C G T A G T C A | CHR(?)/Hela-CellCycle-Expression/Homer | 1e-2 | -6.054e+00 | 0.0713 | 247.0 | 38.18% | 13216.3 | 32.82% | motif file (matrix) | svg |
| 13 | A G T C C T G A A T C G A G C T A G C T G A C T A G T C G C T A A C G T C G A T G C A T C G A T A T C G C G T A T A G C G C A T A T G C C G T A | bZIP:IRF(bZIP,IRF)/Th17-BatF-ChIP-Seq(GSE39756)/Homer | 1e-2 | -5.546e+00 | 0.1093 | 196.0 | 30.29% | 10300.3 | 25.58% | motif file (matrix) | svg |
| 14 | T C A G A G C T A T G C C G T A A G T C T C A G A C G T A T C G T C G A A G T C G A T C T G A C | TFE3(bHLH)/MEF-TFE3-ChIP-Seq(GSE75757)/Homer | 1e-2 | -5.484e+00 | 0.1093 | 61.0 | 9.43% | 2674.4 | 6.64% | motif file (matrix) | svg |
| 15 | T C A G G A C T C A G T C T G A A G C T C T A G G A C T T G C A C T G A A G T C | HLF(bZIP)/HSC-HLF.Flag-ChIP-Seq(GSE69817)/Homer | 1e-2 | -5.425e+00 | 0.1093 | 273.0 | 42.19% | 14941.4 | 37.11% | motif file (matrix) | svg |
| 16 | C T A G G T A C A G T C T G C A A G T C C T G A A G T C A G T C A G T C G C T A | Klf4(Zf)/mES-Klf4-ChIP-Seq(GSE11431)/Homer | 1e-2 | -5.382e+00 | 0.1093 | 296.0 | 45.75% | 16355.9 | 40.62% | motif file (matrix) | svg |
| 17 | T A C G T A C G G T A C A T C G T A C G T A C G G T C A C T G A C G T A G A C T | E2F4(E2F)/K562-E2F4-ChIP-Seq(GSE31477)/Homer | 1e-2 | -5.288e+00 | 0.1093 | 338.0 | 52.24% | 18969.4 | 47.11% | motif file (matrix) | svg |
| 18 | G A C T G C A T C T A G C G A T G A T C T C G A C A T G G A T C | Tgif1(Homeobox)/mES-Tgif1-ChIP-Seq(GSE55404)/Homer | 1e-2 | -5.259e+00 | 0.1093 | 613.0 | 94.74% | 37073.1 | 92.07% | motif file (matrix) | svg |
| 19 | T C A G T A C G T A G C A C G T A C T G C G A T A G T C C G T A T A C G A G T C | Meis1(Homeobox)/MastCells-Meis1-ChIP-Seq(GSE48085)/Homer | 1e-2 | -4.700e+00 | 0.1742 | 544.0 | 84.08% | 32369.9 | 80.39% | motif file (matrix) | svg |
