# Supplementary material for: Mouse strain-specific polymorphic provirus functions as cis-regulatory element leading to epigenomic and transcriptomic variations
Source: Nat Commun. 2021 Nov 9;12:6462. doi: 10.1038/s41467-021-26630-z (PMC8578388; doi:10.1038/s41467-021-26630-z)
Supplement: Supplementary file 8 — Source data [file 41467_2021_26630_MOESM8_ESM.zip › Source Data/Source Data_Figure S2d_quantative file of Western blot.zip!/Source Data_Figure S2d_quantative file of Western blot/FigS2d-2020-07-13 21hr 00min 38sec-report.pdf]

Image Report: 2020-07-13 21hr 00min 38sec

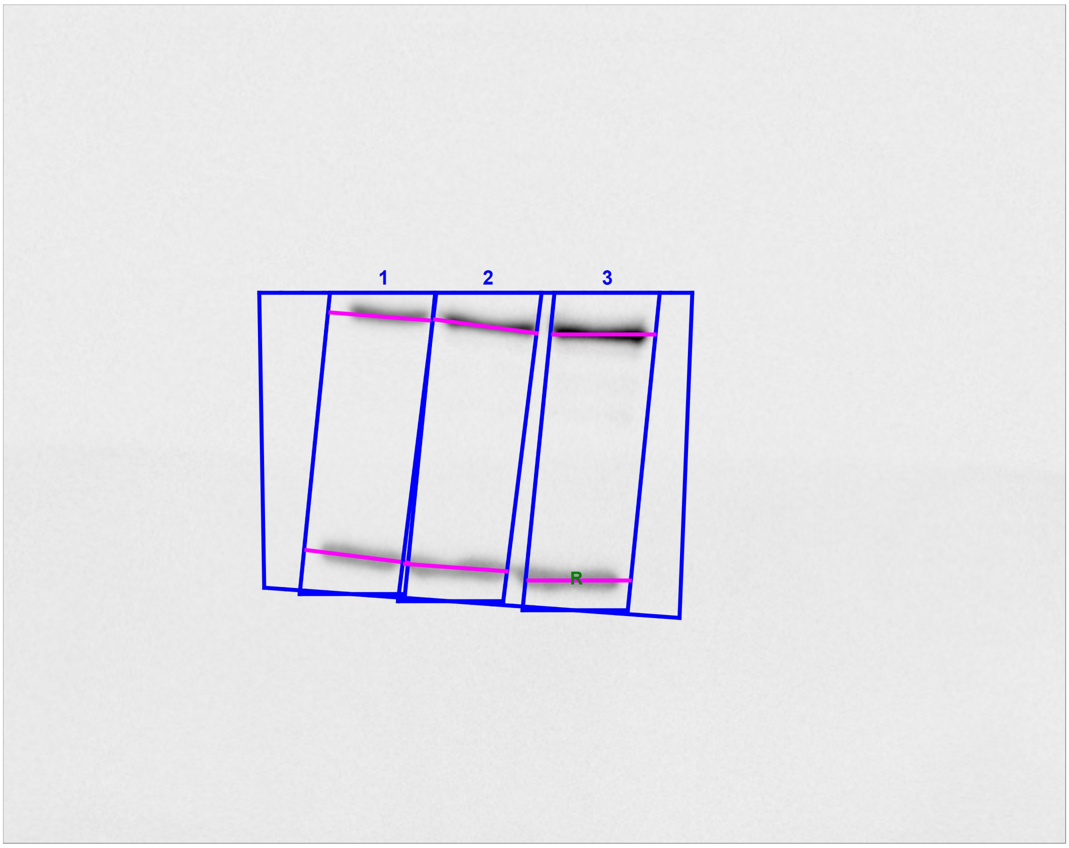

C:\Users\Leung Lab\Downloads\x\2020-07-13 21hr 00min 38sec.scn

Acquisition Information

Imager	ChemiDoc™ Touch
Exposure Time (sec)	11.204 (Signal Accumulation)
Serial Number	732BR0380
Software Version	1.2.0.12
Application	Chemiluminescence
Excitation Source	No Illumination
Emission Filter	No Filter
Binning	2x2

Image Information

Acquisition Date	13/7/2020 9:00:38 PM
User Name	
Image Area (mm)	X: 77.1 Y: 60.8
Pixel Size (μm)	X: 144.0 Y: 144.0
Data Range (Int)	500 - 26973

Analysis Settings

Detection	Lane detection:<br>Automatically detected lanes with manual adjustments<br><br>Band detection:<br>Automatically detected bands with sensitivity: Low<br><br>Lane Background Subtraction:<br>Lane background subtracted with disk size: 10<br><br>Lane width: 7.63 mm
Quantity Analysis	Reference Band: Lane 3 Band 2

Lane Statistics

Lane No.	Adj. Total Band Vol. (Int)	Total Band Vol. (Int)	Adj. Total Lane Vol. (Int)	Total Lane Vol. (Int)	Bkgd. Vol. (Int)	Norm. Factor
1	4,148,151	5,597,595	4,377,164	9,910,735	5,533,571	N/A
2	6,000,925	7,457,683	6,216,211	12,019,764	5,803,553	N/A
3	9,267,474	10,921,074	9,682,199	15,764,108	6,081,909	N/A

Lane And Band Analysis

Lane 1

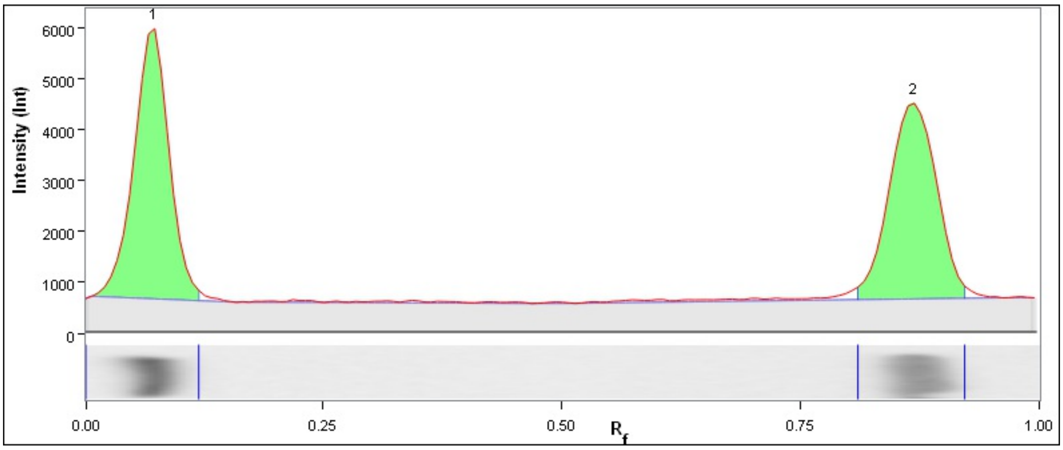

Band No.	Band Label	Mol. Wt. (KDa)	Relative Front	Adj. Volume (Int)	Volume (Int)	Abs. Quant.	Rel. Quant.	Band %	Lane %
1		N/A	0.079	2,097,634	2,847,478	N/A	0.78	50.6	47.9
2		N/A	0.875	2,050,517	2,750,117	N/A	0.76	49.4	46.8

Band Detection	Automatically detected bands with sensitivity: Low
Lane Background	Lane background subtracted with disk size: 10
Lane Width	7.63 mm

Lane 2

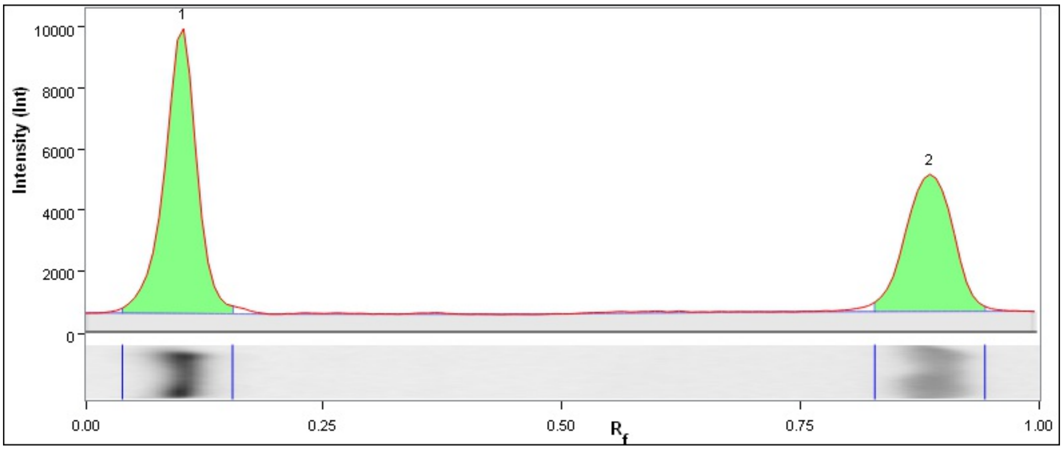

Band No.	Band Label	Mol. Wt. (KDa)	Relative Front	Adj. Volume (Int)	Volume (Int)	Abs. Quant.	Rel. Quant.	Band %	Lane %
1		N/A	0.109	3,523,705	4,213,924	N/A	1.31	58.7	56.7
2		N/A	0.891	2,477,220	3,243,759	N/A	0.92	41.3	39.9

Band Detection	Automatically detected bands with sensitivity: Low
Lane Background	Lane background subtracted with disk size: 10
Lane Width	7.63 mm

Lane 3

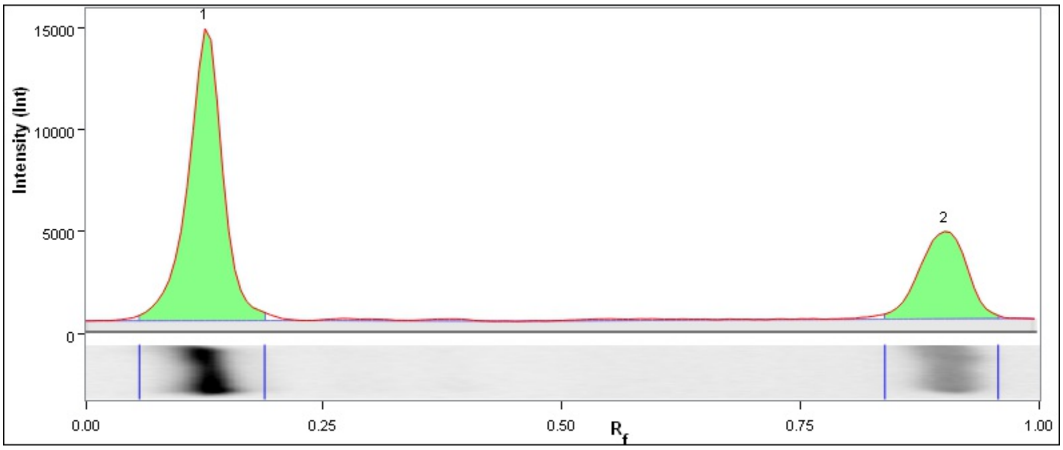

Band No.	Band Label	Mol. Wt. (KDa)	Relative Front	Adj. Volume (Int)	Volume (Int)	Abs. Quant.	Rel. Quant.	Band %	Lane %
1		N/A	0.131	6,573,590	7,381,363	N/A	2.44	70.9	67.9
2		N/A	0.906	2,693,884	3,539,711	N/A	1.00	29.1	27.8

Band Detection	Automatically detected bands with sensitivity: Low
Lane Background	Lane background subtracted with disk size: 10
Lane Width	7.63 mm
