# Supplementary material for: Mouse strain-specific polymorphic provirus functions as cis-regulatory element leading to epigenomic and transcriptomic variations
Source: Nat Commun. 2021 Nov 9;12:6462. doi: 10.1038/s41467-021-26630-z (PMC8578388; doi:10.1038/s41467-021-26630-z)
Supplement: Supplementary file 8 — Source data [file 41467_2021_26630_MOESM8_ESM.zip › Source Data/Source Data_Figure S2d_quantative file of Western blot.zip!/Source Data_Figure S2d_quantative file of Western blot/FigS2d2020-10-31 19hr 21min 37sec-report1.pdf]

Image Report: 2020-10-31 19hr 21min 37sec

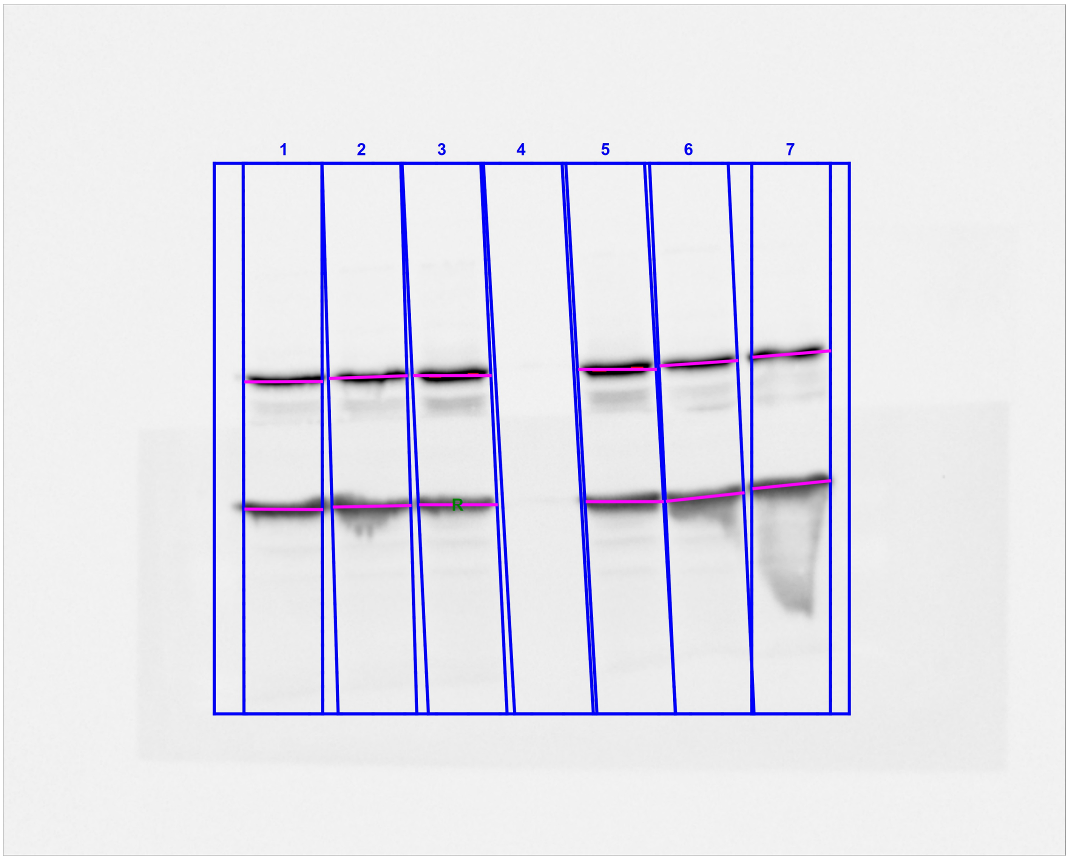

Z:\DL\Chemidoc Touch Images 2020-10-31_19.24.20\2020-10-31 19hr 21min 37sec.scn

Acquisition Information

Imager	ChemiDoc™ Touch
Exposure Time (sec)	1.000 (Signal Accumulation)
Serial Number	732BR0380
Software Version	1.2.0.12
Application	Chemiluminescence
Excitation Source	No Illumination
Emission Filter	No Filter
Binning	4x4

Image Information

Acquisition Date	31/10/2020 7:21:37 PM
User Name	
Image Area (mm)	X: 105.6 Y: 84.6
Pixel Size (μm)	X: 153.2 Y: 153.2
Data Range (Int)	500 - 65535

Analysis Settings

Detection	Lane detection:<br>Automatically detected lanes with manual adjustments<br><br>Band detection:<br>Automatically detected bands with sensitivity: Low<br><br>Lane Background Subtraction:<br>Lane background subtracted with disk size: 10<br><br>Lane width: 7.81 mm
Quantity Analysis	Reference Band: Lane 3 Band 2

Lane Statistics

Lane No.	Adj. Total Band Vol. (Int)	Total Band Vol. (Int)	Adj. Total Lane Vol. (Int)	Total Lane Vol. (Int)	Bkgd. Vol. (Int)	Norm. Factor
1	21,297,651	23,412,315	22,913,892	39,736,599	16,822,707	N/A
2	22,364,673	24,973,527	24,288,954	41,659,095	17,370,141	N/A
3	24,160,638	26,620,266	26,927,745	44,941,965	18,014,220	N/A
4	N/A	N/A	790,959	16,277,823	15,486,864	N/A
5	25,997,811	28,643,181	28,841,010	47,002,671	18,161,661	N/A
6	22,759,005	25,335,015	24,977,046	42,569,190	17,592,144	N/A
7	21,601,713	24,747,087	26,212,164	48,114,063	21,901,899	N/A

Lane And Band Analysis

Lane 1

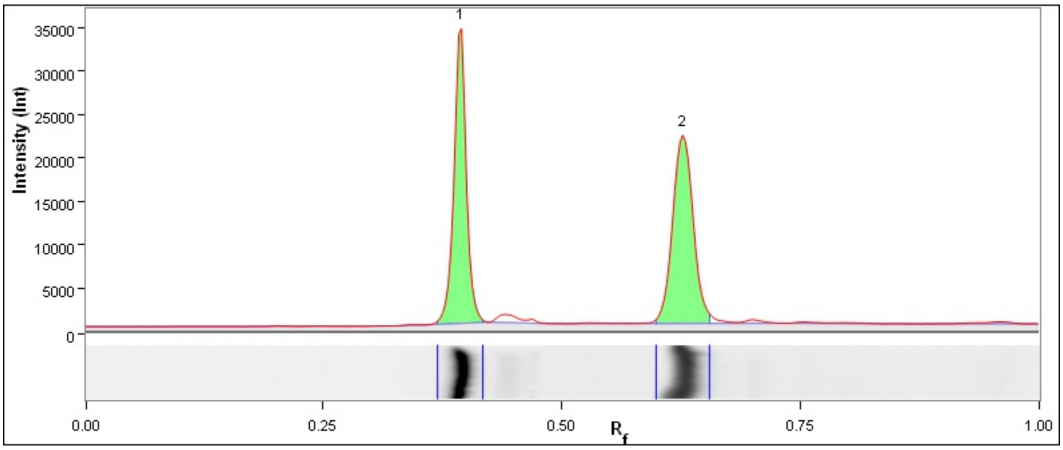

Band No.	Band Label	Mol. Wt. (KDa)	Relative Front	Adj. Volume (Int)	Volume (Int)	Abs. Quant.	Rel. Quant.	Band %	Lane %
1		N/A	0.397	10,326,276	11,308,332	N/A	1.17	48.5	45.1
2		N/A	0.628	10,971,375	12,103,983	N/A	1.24	51.5	47.9

Band Detection	Automatically detected bands with sensitivity: Low
Lane Background	Lane background subtracted with disk size: 10
Lane Width	7.81 mm

Lane 2

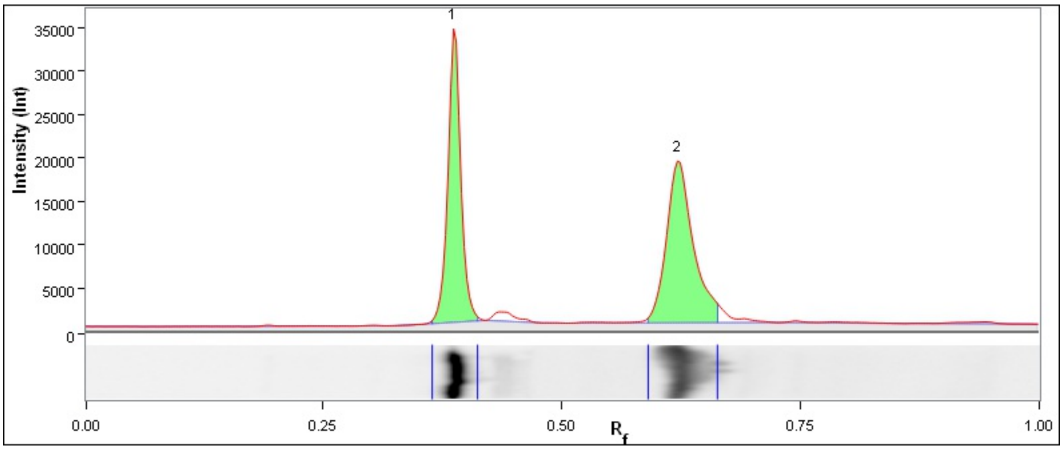

Band No.	Band Label	Mol. Wt. (KDa)	Relative Front	Adj. Volume (Int)	Volume (Int)	Abs. Quant.	Rel. Quant.	Band %	Lane %
1		N/A	0.388	10,404,000	11,486,118	N/A	1.18	46.5	42.8
2		N/A	0.623	11,960,673	13,487,409	N/A	1.36	53.5	49.2

Band Detection	Automatically detected bands with sensitivity: Low
Lane Background	Lane background subtracted with disk size: 10
Lane Width	7.81 mm

Lane 3

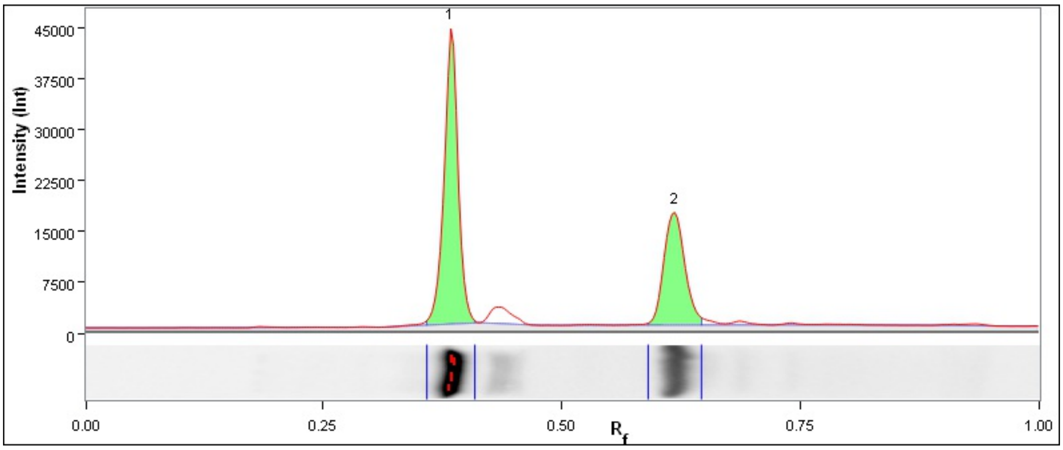

Band No.	Band Label	Mol. Wt. (KDa)	Relative Front	Adj. Volume (Int)	Volume (Int)	Abs. Quant.	Rel. Quant.	Band %	Lane %
1		N/A	0.385	15,337,281	16,596,777	N/A	1.74	63.5	57.0
2		N/A	0.620	8,823,357	10,023,489	N/A	1.00	36.5	32.8

Band Detection	Automatically detected bands with sensitivity: Low
Lane Background	Lane background subtracted with disk size: 10
Lane Width	7.81 mm

Lane 4

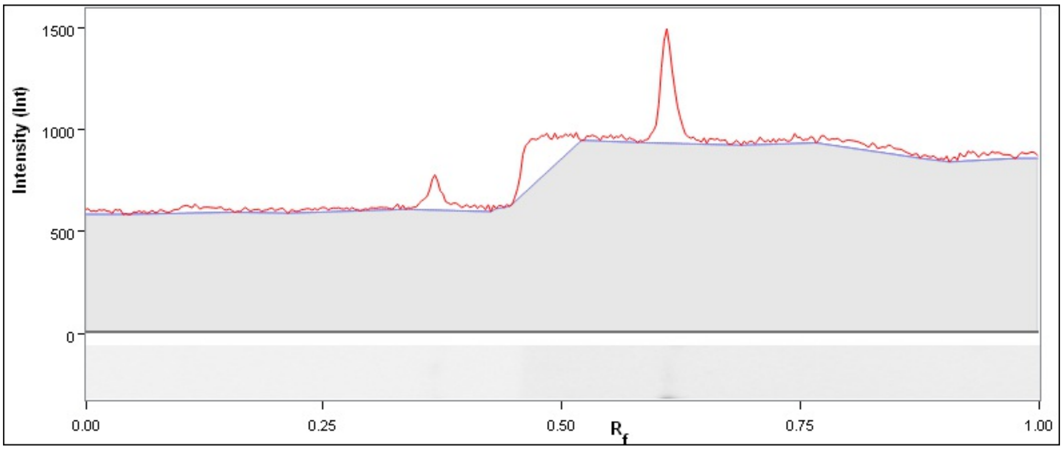

Band No.	Band Label	Mol. Wt. (KDa)	Relative Front	Adj. Volume (Int)	Volume (Int)	Abs. Quant.	Rel. Quant.	Band %	Lane %

Band Detection	Automatically detected bands with sensitivity: Low
Lane Background	Lane background subtracted with disk size: 10
Lane Width	7.81 mm

Lane 5

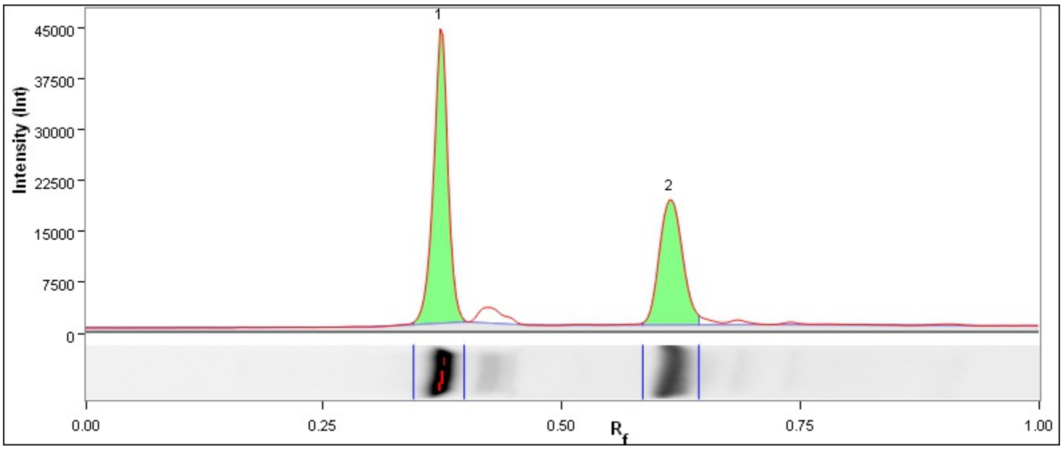

Band No.	Band Label	Mol. Wt. (KDa)	Relative Front	Adj. Volume (Int)	Volume (Int)	Abs. Quant.	Rel. Quant.	Band %	Lane %
1		N/A	0.374	15,453,765	16,849,380	N/A	1.75	59.4	53.6
2		N/A	0.615	10,544,046	11,793,801	N/A	1.20	40.6	36.6

Band Detection	Automatically detected bands with sensitivity: Low
Lane Background	Lane background subtracted with disk size: 10
Lane Width	7.81 mm

Lane 6

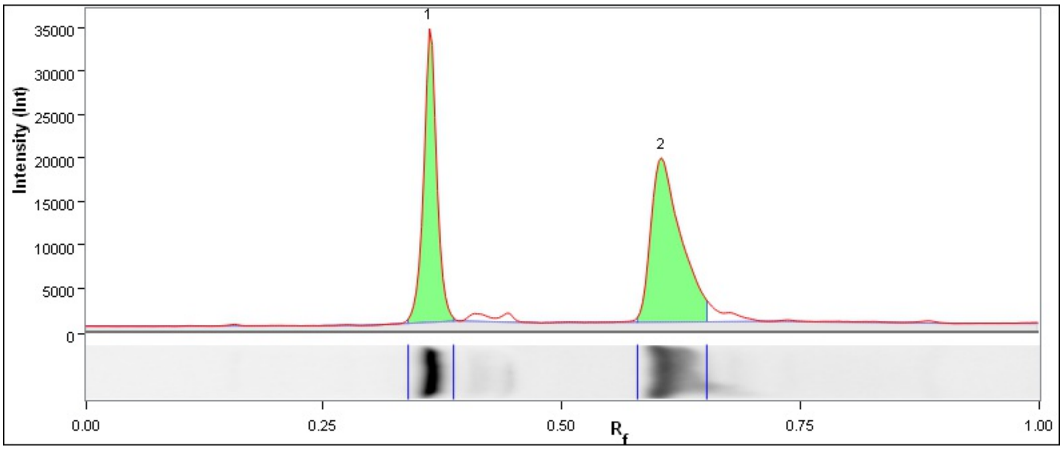

Band No.	Band Label	Mol. Wt. (KDa)	Relative Front	Adj. Volume (Int)	Volume (Int)	Abs. Quant.	Rel. Quant.	Band %	Lane %
1		N/A	0.363	9,979,323	10,994,019	N/A	1.13	43.8	40.0
2		N/A	0.606	12,779,682	14,340,996	N/A	1.45	56.2	51.2

Band Detection	Automatically detected bands with sensitivity: Low
Lane Background	Lane background subtracted with disk size: 10
Lane Width	7.81 mm

Lane 7

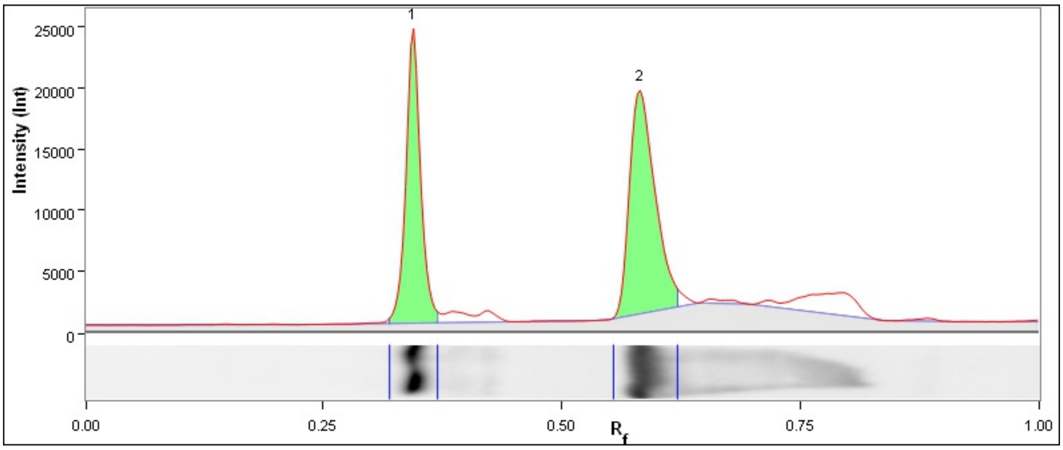

Band No.	Band Label	Mol. Wt. (KDa)	Relative Front	Adj. Volume (Int)	Volume (Int)	Abs. Quant.	Rel. Quant.	Band %	Lane %
1		N/A	0.346	9,315,609	10,122,837	N/A	1.06	43.1	35.5
2		N/A	0.584	12,286,104	14,624,250	N/A	1.39	56.9	46.9

Band Detection	Automatically detected bands with sensitivity: Low
Lane Background	Lane background subtracted with disk size: 10
Lane Width	7.81 mm
